# A 3D Bioprinted Platform That Maintains the Functional Integrity of Primary AML Cells

**DOI:** 10.1111/cpr.70250

**Published:** 2026-06-16

**Authors:** Wanling Huang, Bo Deng, Nini Guo, Yiyi Ding, Zhangjingwen Xu, Yiwen Lu, Yue Ma, Qian Ren, Nan Wang, Pengyu Huang, Xiaotong Ma

**Affiliations:** ^1^ State Key Laboratory of Experimental Hematology, National Clinical Research Center for Blood Diseases, Institute of Hematology & Blood Diseases Hospital Chinese Academy of Medical Sciences & Peking Union Medical College Tianjin China; ^2^ Tianjin Institutes of Health Science Tianjin China; ^3^ State Key Laboratory of Advanced Medical Materials and Devices, Engineering Research Center of Pulmonary and Critical Care Medicine Technology and Device (Ministry of Education), Tianjin Key Laboratory of Biomedical Materials, Institute of Biomedical Engineering Chinese Academy of Medical Science & Peking Union Medical College Tianjin China

**Keywords:** 3D bioprinting, acute myeloid leukaemia, drug screening, metabolism

## Abstract

Acute myeloid leukaemia (AML) urgently requires more reliable in vitro platforms for drug evaluation, as existing models often fail to maintain the phenotypic, metabolic, and pharmacologic features of primary leukaemic cells. To address this limitation, we developed a rapidly assembled, screening‐compatible three‐dimensional (3D) bioprinting platform. It encapsulates patient‐derived bone marrow mononuclear cells within a gelatin‐hyaluronic acid hydrogel, whose mechanical properties are tuned to match those of native bone marrow. Within this controlled 3D microenvironment, primary AML cells better maintain an in vivo‐like state, showing enhanced viability, sustained proliferative capacity, preservation of stem‐like subpopulations, and drug responses that more closely mirror clinical behaviour. Transcriptomic profiling further revealed robust activation of MYC target programs and mTORC1 signalling, accompanied by elevated oxidative phosphorylation and glycolytic activity, indicative of the highly proliferative and metabolically active state exhibited by AML cells in vivo. These findings demonstrate that our patient‐specific, 3D bioprinted system provides a high‐fidelity model. This model faithfully recapitulates AML physiology and metabolic features while capturing inter‐patient variability. Consequently, it offers a more reliable and predictive platform for preclinical drug assessment.

## Graphical abstract

This study presents a 3D bioprinted platform using gelatin‐hyaluronic acid hydrogels to enhance primary AML cell survival and proliferation while preserving stem‐like properties in vitro. By recapitulating in vivo‐like metabolic activity and drug resistance, this system offers a robust tool for preclinical drug screening and personalised therapy.

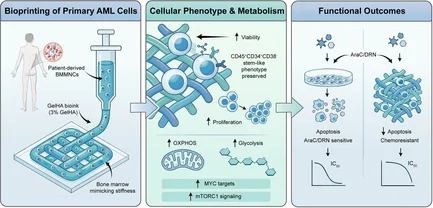

## 1 Introduction

Acute myeloid leukaemia (AML) is characterised by profound heterogeneity and aggressive clinical behaviour [1]. Despite advances in molecular pathogenesis, the standard ‘7 + 3’ induction regimen has remained largely unchanged for decades [2]. Although standard chemotherapy induces complete remission in approximately half of adult patients, only 10%–20% achieve durable leukaemia‐free survival, and outcomes are even worse for those with relapsed or refractory disease [3, 4, 5]. These clinical observations underscore the intrinsic resistance and therapeutic vulnerability that define AML.

Conventional preclinical approaches, such as two‐dimensional (2D) culture systems and mouse xenograft models, have long supported studies of leukaemic cell signalling, clonal dynamics and therapeutic responses. Yet these platforms fall short of capturing the architectural complexity, extracellular matrix cues, and metabolic heterogeneity that characterize human bone marrow. Simple monolayer cultures cannot recreate the supportive stromal interactions or the physiologic gradients in oxygen, nutrients, and cytokines that influence stemness and cell fate [6, 7]. Mouse models offer valuable in vivo context but are inherently limited by interspecies differences in cytokine networks and metabolism, which reduces their ability to predict clinical outcomes. As a result, numerous compounds that perform well in murine systems fail to translate into human efficacy [8]. Primary human AML cells represent the most physiologically relevant model, yet their application is constrained by limited sample availability and the inability of conventional 2D cultures to maintain leukaemic identity, viability and stemness [9, 10]. Moreover, the requirement for exogenous cytokines to sustain leukaemic stem cells inevitably perturbs their native functional states. Collectively, these limitations likely contribute to the slow progress in novel drug development and personalised treatment strategies for patients.

Recent advances in three‐dimensional (3D) culture systems and organoid technologies provide transformative opportunities to overcome these limitations [6, 11]. By better preserving nutrient gradients, oxygen tension, mechanical cues, and cell‐matrix interactions [11, 12, 13], 3D culture systems create a microenvironment that allows AML cells to survive, proliferate, and function in a manner that more closely reflects in vivo biology [7, 14]. Unlike traditional 3D culture systems that rely on spontaneous cell aggregation or matrix‐gel self‐assembly, 3D bioprinting enables programmable, spatially defined placement of cells, matrix components and bioactive factors [15]. Through computer‐aided design (CAD), the printed constructs can be engineered with precisely controlled geometry, porosity, fluidic channels, and uniform cell distribution, thereby greatly improving reproducibility and standardisation of in vitro models. The mechanical stiffness, viscoelasticity, and degradability of bioinks can be finely tuned to mimic specific tissue microenvironments [16], such as the bone marrow niche, which is particularly critical for AML cells [17]. Previous studies have demonstrated that 3D‐printed matrices with adjustable mechanical properties can influence hematologic cancer cell proliferation, drug resistance and stemness maintenance [18, 19, 20].

Importantly, in April 2025, the U.S. Food and Drug Administration announced a strategic initiative to gradually phase out animal testing requirements for monoclonal antibodies and other therapeutics, encouraging the adoption of ‘new alternative methodologies’, including organoids, organ‐on‐chip platforms and real‐world evidence. This policy shift, alongside ongoing limitations of existing preclinical models, underscores an urgent need to develop more human‐relevant, predictive in vitro systems for drug development [21]. In this context, we aimed to establish a robust 3D culture platform for primary AML samples. We hypothesised that such a system could better preserve the patient‐specific disease biology in vitro, with the long‐term goal of enhancing translational predictability for therapeutic screening and reducing the reliance on animal experimentation.

In this study, we evaluated for the first time whether 3D bioprinting can be applied to primary AML bone marrow mononuclear cells (BMMNCs) to advance physiologically relevant in vitro models of AML. We assessed the printability of primary AML cells, optimised the bioprinting strategy, and established standardised protocols for downstream analyses. Our results show that primary AML cells can be efficiently bioprinted in 3D, exhibiting improved viability, metabolic state and drug resistance without the need for exogenous stimuli or stromal support. Together, these findings establish an innovative and reproducible 3D culture platform for leukaemia research.

## 2 Materials and Methods

### 2.1 Patient Samples

Bone marrow specimens were obtained from patients with diagnosed AML at the Institute of Haematology and Blood Diseases Hospital, Chinese Academy of Medical Sciences, after informed consent. All experiments were conducted in accordance with institutional ethical guidelines and approved by the Ethics Committee of the Institute of Haematology and Blood Diseases Hospital.

Inclusion criteria were as follows: (1) patients with a confirmed diagnosis of AML based on clinical and pathological evaluation; (2) age ≥ 18 years; (3) availability of complete clinical information; (4) sufficient viable cells in the collected samples for experiments and (5) approval from the institutional ethics committee and acquisition of informed consent from all patients. Exclusion criteria included: (1) the presence of concurrent malignancies of other origins; and (2) co‐existing haematological disorders. Clinical and genetic characteristics of the AML samples used in this study are summarised in Table 1.

**TABLE 1:** Characteristics of primary AML samples.

Patient	AML#1	AML#2	AML#3
Age/gender	52 years/male	46 years/male	58 years/female
Diagnosis	De novo	Relapse	Relapse
Cytogenetics	Normal	44, XY, del(5) (q15q35), −7, der(13;15) (q10;q10) [8]	Normal
Mutations	NRAS	U2AF1	SRSF2, DNMT3A
Immunopheno‐typing	CD7^+^ CD34^+^ CD38^+^ CD33^+^ CD117^low^ CD13^low^ HLA‐DR^low^	CD117^+^ CD38^+^ CD33^+^ CD13^low^ MPO^low^ CD34^−^ HLA‐DR^−^ CD123^−^ CD56^−^ CD7^−^ CD15^−^ CD11b^−^ CD16^−^CD64^−^CD4^−^CD14^−^	CD117^++^ CD34^+^ CD13^+^ CD33^+^ HLA‐DR^+^ CD38^low^ CD4^low^ CD123^low^ MPO^−^ CD56^−^ CD7^−^
Abnormal cells (% of nucleated cells)	53%	92%	78%

### 2.2 3D Bioprinting of the AML Model

Primary BMMNCs were freshly isolated from patient bone marrow aspirates and processed for bioprinting. To generate a bioink capable of recapitulating the biomechanical and architectural features of the bone marrow niche, AML cells were gently suspended in GelHA bioink consisting of gelatin methacrylate (GelMA, SP‐BI‐G01‐4, SUNP Biotech) and hyaluronic acid (HA, H131007, Aladdin) at a 1:1 ratio, supplemented with a photoinitiator. The bioink was maintained on ice to preserve low viscosity and ensure homogeneous cell distribution. Based on optimisation studies, a 3% GelHA formulation was selected as it provided an optimal balance of printability, mechanical stability and cell compatibility.

Bioprinting was performed using a computer‐assisted extrusion bioprinter (SUNPBIOMAKER2i, SUNP). The AML‐GelHA bioink mixture was extruded through a fine‐gauge nozzle under controlled pressure and deposited layer by layer to generate grid‐like 3D constructs with defined architecture. Each printed layer was immediately photocrosslinked under UV illumination to stabilise the structure. Following printing, the 3D AML constructs were transferred to culture plates and maintained under standard conditions (37°C, 5% CO_2_).

### 2.3 Drugs

Cytarabine (HY‐13605, MedChemExpress) and daunorubicin (HY‐13062, MedChemExpress) were dissolved in PBS. 10058‐F4 (HY‐12702, MedChemExpress, 100 μM) and rapamycin (HY‐10219, HY‐10219, 80 nM) were dissolved in PBS containing 5% DMSO, aliquoted, and stored at −80°C until use.

### 2.4 Flow Cytometry Analysis

Cells cultured in 3D constructs were degraded from GelHA using a GelHA‐specific lysis solution (SUNP Biotech) and collected by centrifugation to obtain a single‐cell suspension. Cells were stained with fluorochrome‐conjugated antibodies against CD45 (PE, HI30, BD Biosciences), CD33 (APC, WM53, Biolegend), CD34 (FITC, 581, Biolegend) and CD38 (PerCP‐Cy5.5, HIT2, BD Biosciences). Flow cytometry was performed on a FACS Canto II or LSRFortessa (BD Biosciences). Data were analysed using FACSDiva (BD Biosciences) or FlowJo software (Tree Star).

### 2.5 Cell Apoptosis and Cell Cycle Analysis

Primary AML BMMNCs were treated with AraC (4 or 65 nM) or daunorubicin (3 or 7 nM) for 72 h. Apoptosis was assessed using Annexin V and 7‐AAD staining (Annexin V Apoptosis Detection Kit; BD Biosciences) followed by flow cytometry. For cell cycle analysis, cells were fixed, permeabilized, and stained with PI (BD Biosciences) prior to flow cytometric assessment.

### 2.6 Cell Survival Assessment

To evaluate the effects of bioprinting and early culture on cell viability, AML‐laden constructs were cultured for 8 days at 37°C and 5% CO_2_. Cell survival was assessed using a fluorescence‐based Live/Dead assay. Constructs were rinsed with PBS and incubated with a staining solution containing Calcein‐AM and propidium iodide (PI) (Beyotime) for 30 min at 37°C. Following gentle PBS washing, constructs were imaged using a fluorescence microscope to visualise live and dead cells. Cell survival was quantified using ImageJ as the percentage of live cells relative to total cells.

### 2.7 Quantification of Cell Numbers in 2D and 3D Cultures

To compare growth dynamics between 2D and 3D cultures, cell numbers were measured at days 0, 2, 4, 6 and 8. For 3D constructs, GelHA hydrogels were fully degraded using a GelHA‐specific lysis solution (SUNP Biotech), and the released cells were collected and counted. Cells from parallel 2D cultures were harvested and counted at the same time points. Cell numbers were used to generate proliferation curves.

### 2.8 Mechanical Testing of GelHA Hydrogels

The Young's modulus of GelHA hydrogels at various concentrations was measured using a nanoindenter. Photocrosslinked GelHA samples were equilibrated in PBS and mechanically tested under controlled loading conditions.

### 2.9 PCR Amplification and Sanger Sequencing

Genomic DNA (gDNA) was extracted from primary AML cells after 6 days of culture in both 2D conditions and 3D bioprinted constructs using the TIANamp Genomic DNA Kit (YDP304, Tiangen), according to the manufacturer's instructions. Target regions were amplified by PCR using the BigDye Terminator v3.1 Cycle Sequencing Kit (Thermo), followed by sequencing on an ABI 3730XL DNA Analyser. Primer sequences are listed in Table S1.

### 2.10 Bulk RNA Sequencing

Total RNA was extracted from patient‐derived AML cells cultured under conventional 2D conditions or within 3D GelHA constructs. RNA integrity was assessed using a Bioanalyzer 2100 (Agilent). mRNA libraries were prepared through mRNA enrichment, fragmentation, cDNA synthesis, adapter ligation, and PCR amplification and then sequenced on an Illumina platform with paired‐end reads. Clean reads were aligned to the human reference genome (GRCh38) using HISAT2, and gene expression was quantified with featureCounts. Differential expression and pathway analyses—including KEGG and Gene Set Enrichment Analysis (GSEA)—were performed using clusterProfiler to compare transcriptional programs and microenvironment‐driven regulatory networks between 2D and 3D AML cultures.

### 2.11 Quantitative Real‐Time PCR

RNA was isolated using TRIzol (Thermo Fisher Scientific) and reverse‐transcribed using the TransScript All‐in‐One First‐Strand cDNA Synthesis SuperMix (TransGen Biotech). Quantitative PCR was performed using SYBR Green Premix Pro Taq HS (Takara) on an ABI 7500 system. Relative gene expression was normalised to ACTIN. Primer sequences are listed in Table S2.

### 2.12 Seahorse Assays

For Seahorse analysis, cells from the 3D constructs were released by hydrogel digestion as described above. To ensure consistency, cells from 2D cultures were subjected to the same digestion and centrifugation procedures prior to measurement. Oxidative phosphorylation and glycolysis were measured using the Seahorse XFp Cell Mito Stress Test and Glycolysis Stress Test kits (Agilent Technologies), following the manufacturer's instructions. Briefly, 5 × 10^4^ cells per well were seeded onto Cell‐Tak‐coated (Corning) XFe96 plates. One hour before measurement, culture medium was replaced with Seahorse XF assay medium. For mitochondrial respiration, OCR was recorded at baseline and following sequential injection of oligomycin (1 mM), FCCP (1 mM) and rotenone/antimycin A (0.5 mM).For glycolytic capacity, ECAR was measured at baseline and following injection of glucose (10 mM), oligomycin (1 μM) and 2‐deoxy‐glucose (50 mM).

### 2.13 Statistics and Reproducibility

All statistical data analyses were performed using GraphPad Prism v.8 (GraphPad Software). No statistical method was used to predetermine sample size. No data were excluded from the analyses. Given the limited number of replicates, the data were not formally tested for normality. Results are displayed as the mean ± SD. Statistical significance between two groups was assessed using a two‐tailed Student's *t*‐test. Comparisons among more than two groups were performed using one‐way ANOVA followed by Tukey's post hoc test. For the analysis of growth curves, a two‐way ANOVA was utilised. Dose–response curves were fitted using a four‐parameter logistic regression model with a variable slope (Hill slope) to calculate IC_50_ values. Curve fitting was performed using the ‘log(inhibitor) versus normalised response‐variable slope’ model in GraphPad Prism.

## 3 Results

### 3.1 Establishment of a 3D Bioprinted AML Drug Screening Platform Using Patient‐Derived Cells

To recapitulate key features of the bone marrow microenvironment for AML cells in vitro, we established a 3D bioprinted platform that integrates patient‐derived primary AML cells with a mechanically tunable GelHA Bioink, composed of GelMA and HA in a 1:1 ratio (Figure 1A). Primary BMMNCs were gently suspended in the GelHA enriched with a photoinitiator, ensuring uniform cell distribution throughout the bioink prior to printing. Using a computer‐assisted extrusion‐based bioprinting approach, the cell‐GelHA mixture was extruded through a fine nozzle and crosslinked in a layer‐by‐layer mode via controlled UV illumination. This process yielded stable, architecturally defined 3D constructs with high spatial precision, enabling prolonged culture of primary AML cells under conditions that mimic the spatial confinement, ECM composition, and nutrient gradients characteristic of the native marrow niche.

**FIGURE 1:**
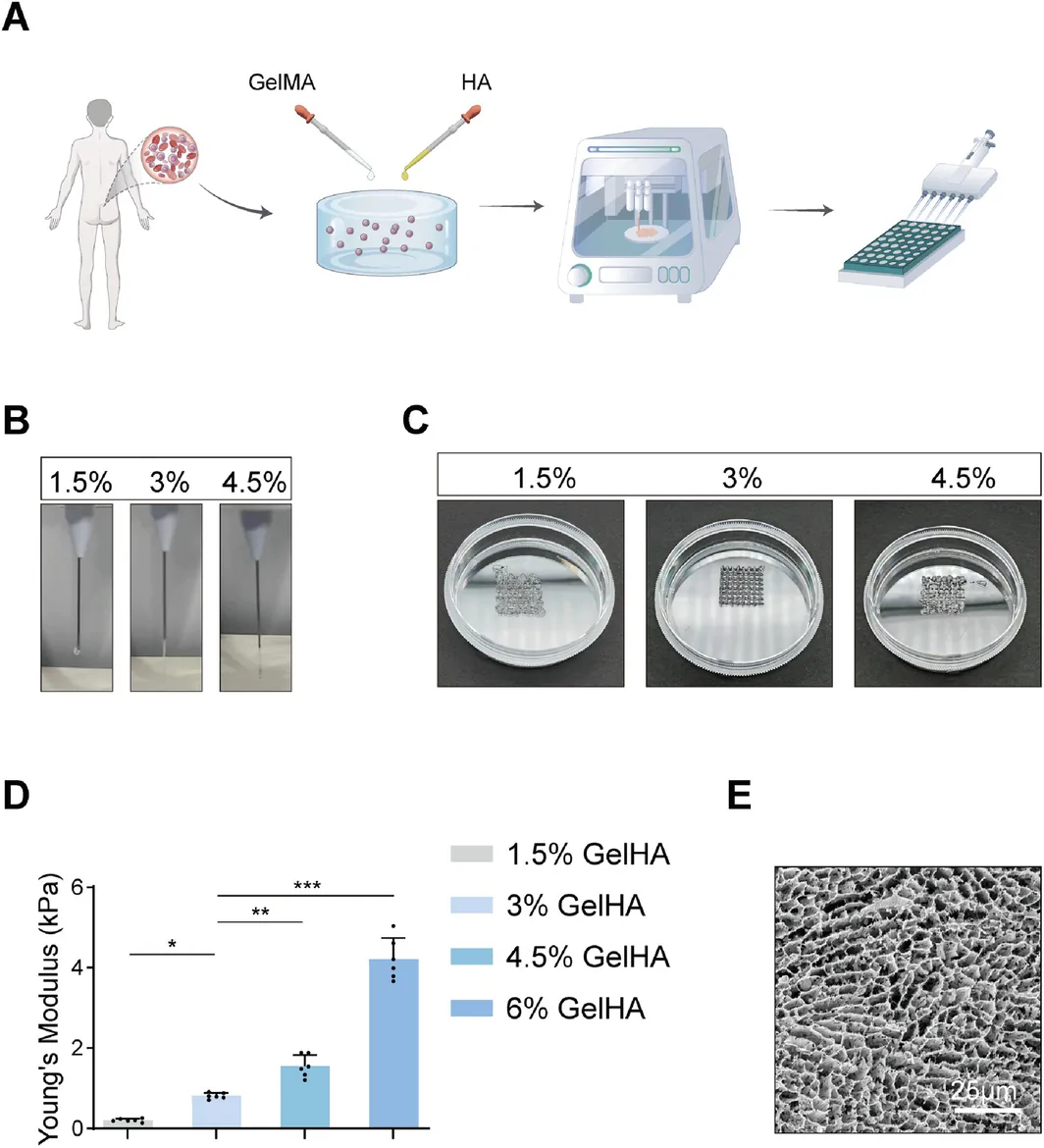
Establishment and optimisation of a 3D bioprinted platform for modelling the AML bone marrow microenvironment. (A) Schematic outline of the 3D bioprinting workflow integrating patient‐derived primary AML blasts with a mechanically tunable GelHA hydrogel. (B) Representative printed constructs using GelHA formulations at 1.5%, 3.0% and 4.5%. (C) The printability and structural performance of scaffolds with a grid‐like structure using different concentrations of the GelHA (1.5%, 3% and 4.5%). (D) Young's modulus measurements of GelHA hydrogels at 1.5%, 3.0%, 4.5% and 6.0% concentrations (*n* = 6 technical replicates). (E) SEM images of scaffolds printed with 3% GelHA. Scale bar, 25 μm. Data are shown as mean ± SD, by one‐way ANOVA (*p < 0.05, **p < 0.01, ***p < 0.001).

Incorporating HA into the GelHA serves multiple purposes. HA is a major component of the native bone marrow extracellular matrix and contributes to viscoelasticity, hydration and cell‐matrix interactions. Its presence promotes AML cell adhesion, migration, and retention, better recapitulating the biochemical cues of the bone marrow microenvironment [22, 23]. Moreover, HA enhances the diffusion of nutrients and signalling molecules while maintaining a compliant matrix that protects fragile primary cells during the bioprinting process. The combination of GelMA and HA therefore creates a microenvironment that is both mechanically and biologically relevant.

To optimise the GelHA formulation and ensure consistent bioprinting performance, we systematically evaluated hydrogels across a range of concentrations (1.5%, 3.0% and 4.5%) (Figure 1B,C). Lower concentrations (1.5%) generated constructs with high initial softness but insufficient structural integrity, resulting in compromised shape retention and reduced reproducibility during printing. Conversely, higher concentrations (4.5%) improved mechanical stiffness and structural fidelity but significantly reduced print smoothness and increased extrusional resistance, leading to frequent nozzle clogging and impaired layer uniformity. In contrast, 3% GelHA offered the most favourable balance between mechanical stability and bioink fluidity, yielding high‐resolution architectural features while maintaining a viscoelastic profile conducive to embedding and protecting fragile primary AML cells.

To further assess the physical relevance of the 3D microenvironment created by the GelHA, we quantified the elastic modulus (Young's modulus) across a broader range of concentrations (1.5%, 3.0%, 4.5% and 6.0%) (Figure 1D). As expected, increasing concentration resulted in progressively stiffer matrices, reflecting enhanced crosslinking density. Notably, the 3% GelHA hydrogel exhibited a modulus within the range reported for native bone marrow tissue, suggesting that this formulation provides a physiologically relevant mechanical milieu for AML cells. This mechanical fidelity is crucial because AML blasts are highly mechanosensitive, and matrix stiffness has been shown to influence leukaemic cell survival, differentiation state, and responses to chemotherapy. Thus, selecting a stiffness‐matched GelHA formulation substantially enhances the biological relevance of downstream functional assays.

Morphological characterisation of the printed hydrogels further confirmed the suitability of the 3% GelHA formulation. Scanning electron microscopy revealed a highly interconnected and uniformly distributed porous network within the matrix (Figure 1E). The pore geometry and spacing are consistent with efficient nutrient and oxygen diffusion, while the compliant ECM‐like architecture, supported by HA, provides ample space to accommodate AML cells, allowing them to proliferate, form aggregates, and maintain disease‐relevant phenotypes.

Collectively, these findings demonstrate that the optimised 3D bioprinted GelHA platform provides a robust, mechanically and biologically relevant microenvironment for patient‐derived AML cells, enabling reliable downstream functional assays and drug‐response profiling.

### 3.2 Characterisation of AML Cells in 3D Culture Conditions

After establishing a stable and structurally defined 3D bioprinted framework, we next sought to determine whether the engineered microenvironment could support the phenotypic integrity and long‐term viability of primary AML cells—key criteria for faithfully modelling disease biology in vitro. Flow cytometric profiling confirmed that the embedded patient‐derived AML cells retained their characteristic immunophenotype, expressing myeloid lineage markers CD45^+^CD33^+^ [24] (Figure 2A). Notably, the primitive CD45^+^CD34^+^CD38^−^ immunophenotype [25, 26] was preserved in 3D culture after 6 days, in contrast to its reduction in 2D conditions (Figures 2B and S1A). In addition, we assessed the impact of 3D bioprinting on the genetic integrity of AML cells. Based on clinical diagnostic reports, we identified AML‐associated mutations in three patient samples included in this study. PCR amplification followed by Sanger sequencing demonstrated that these disease‐associated mutations were stably maintained 6 days after bioprinting, indicating that the process did not alter the underlying genetic features of the leukaemic cells (Figures 2C and S1B). These results indicate that the mechanical and photopolymerisation stresses inherent to bioprinting do not compromise AML cell identity.

**FIGURE 2:**
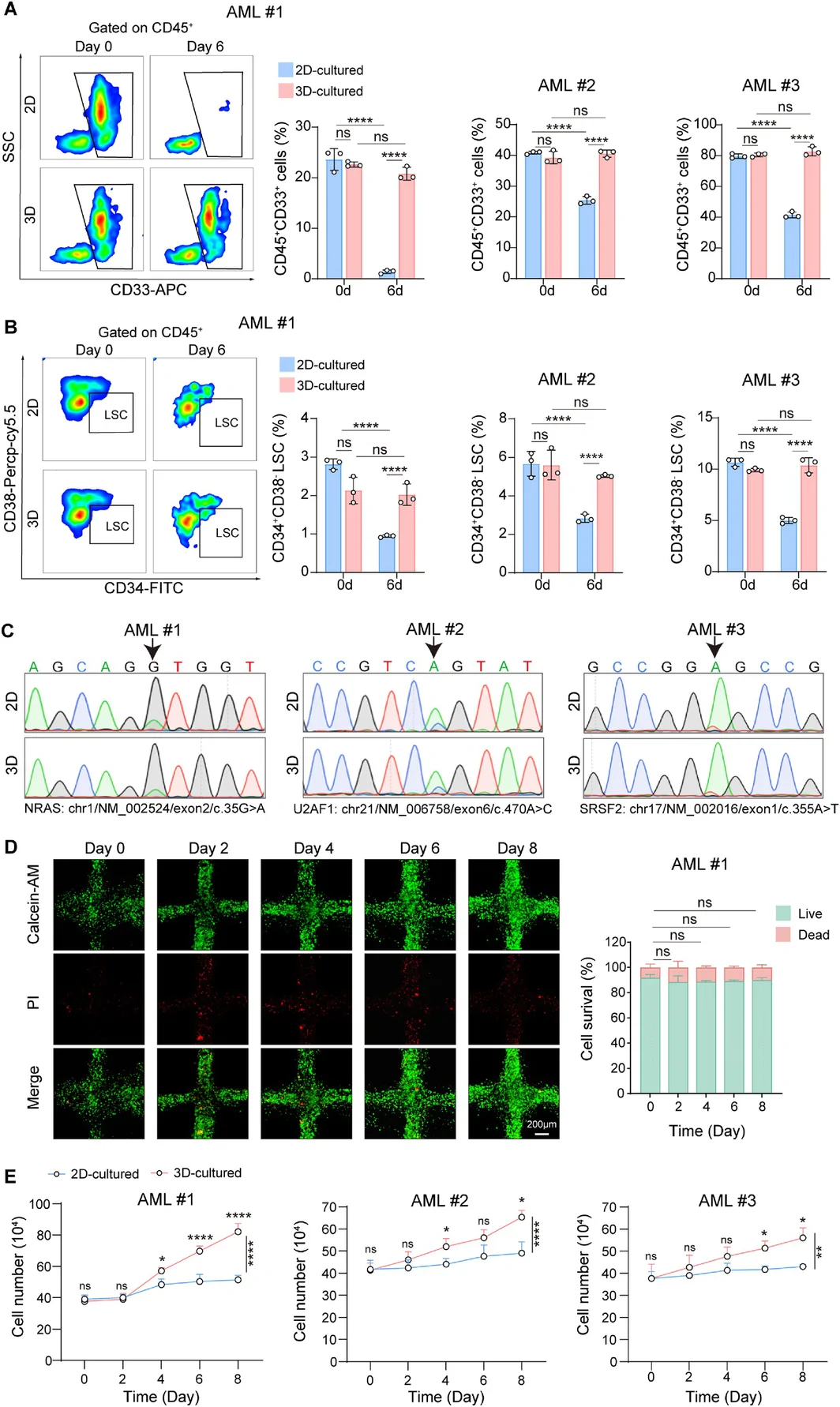
Analysis of AML cell phenotype, viability and proliferation in 3D culture. (A, B) Representative fluorescence‐activated cell sorting (FACS) plots showing CD45^+^CD33^+^ myeloid cells (A) and CD45^+^CD34^+^CD38^−^ LSCs (B) in BMMNCs from the 2D and 3D culture groups at day 0 and day 6, and the corresponding frequencies from three AML patient samples (*n* = 3 technical replicates). (C) Representative Sanger sequencing chromatograms showing AML‐associated hotspot mutations (arrowheads) in genomic DNA extracted from BMMNCs of three AML patients after 6 days of 2D or 3D culture. (D) Representative live/dead staining images and assessment of cell viability of BMMNCs‐laden constructs at days 0, 2, 4, 6, and 8 post‐printing (*n* = 5 technical replicates). Live and dead cells were labelled with Calcein‐AM (green) and PI (red), respectively. Scale bar, 200 μm. (E) Growth curves of BMMNCs from three patients over 8 days under 2D and 3D culture conditions (*n* = 3 technical replicates). Data are shown as mean ± SD, by two‐way ANOVA (A, B, E) or one‐way ANOVA (D) (*p < 0.05, **p < 0.01, ****p < 0.0001, ns, not significant). LSC, leukaemic stem cell; PI, propidium iodide.

We then monitored cell viability and spatial distribution over time to evaluate the platform's capacity to support long‐term culture. Live/dead staining performed at days 0, 2, 4, 6, and 8 after printing revealed consistently high viability, with live cells exhibiting uniform distribution throughout the constructs and minimal evidence of cell clustering or necrotic zones. This homogeneous spatial arrangement suggests effective nutrient diffusion and low shear stress gradients within the printed architecture. Quantitative analysis confirmed that live cell proportions remained above 85% at all measured time points, demonstrating that the bioprinting process and long‐term culture conditions are well‐tolerated by primary AML blasts (Figures 2D and S1C,D).

To further assess AML cell survival in the 3D system, we quantified total cell numbers over an 8‐day culture period. Typically, primary BMMNCs do not expand during prolonged in vitro culture; strikingly, we observed a significant increase in cell counts in the 3D group (Figure 2E). This homeostatic growth pattern reflects the rapid and active proliferation of AML cells within the bone marrow niche in vivo and underscores the ability of the bioprinted microenvironment to recapitulate physiologically relevant disease constraints.

Together, these data demonstrate that the GelHA‐based 3D bioprinted platform preserves AML cell identity across diverse genetic backgrounds while maintaining high viability and proliferation, establishing it as a robust and physiologically relevant model for mechanistic and therapeutic studies.

### 3.3 3D Culture Promotes AML Cell Survival and Proliferation and Activates MYC/mTORC1 Signalling

To identify the genes and pathways affected by the 3D printing strategy, we performed RNA‐seq analysis to compare patients' BMMNCs cultured in our 3D bioprinted system with those in conventional 2D culture. Principal Component Analysis (PCA) revealed clear segregation of samples by culture conditions. Specifically, 2D and 3D samples separated substantially along PC1 (explaining 45.81% of total variance), while the effect of the 3D environment itself was captured along PC2 (explaining 14.25% of the total variance) (Figure 3A). The volcano plot showed that, compared to 2D culture, 2979 genes were upregulated and 2791 were downregulated in 3D‐bioprinted samples (Figure 3B). Similarly, the heatmap (Figure 3C) demonstrated a clear distinction between 2D and 3D samples in terms of gene expression.

**FIGURE 3:**
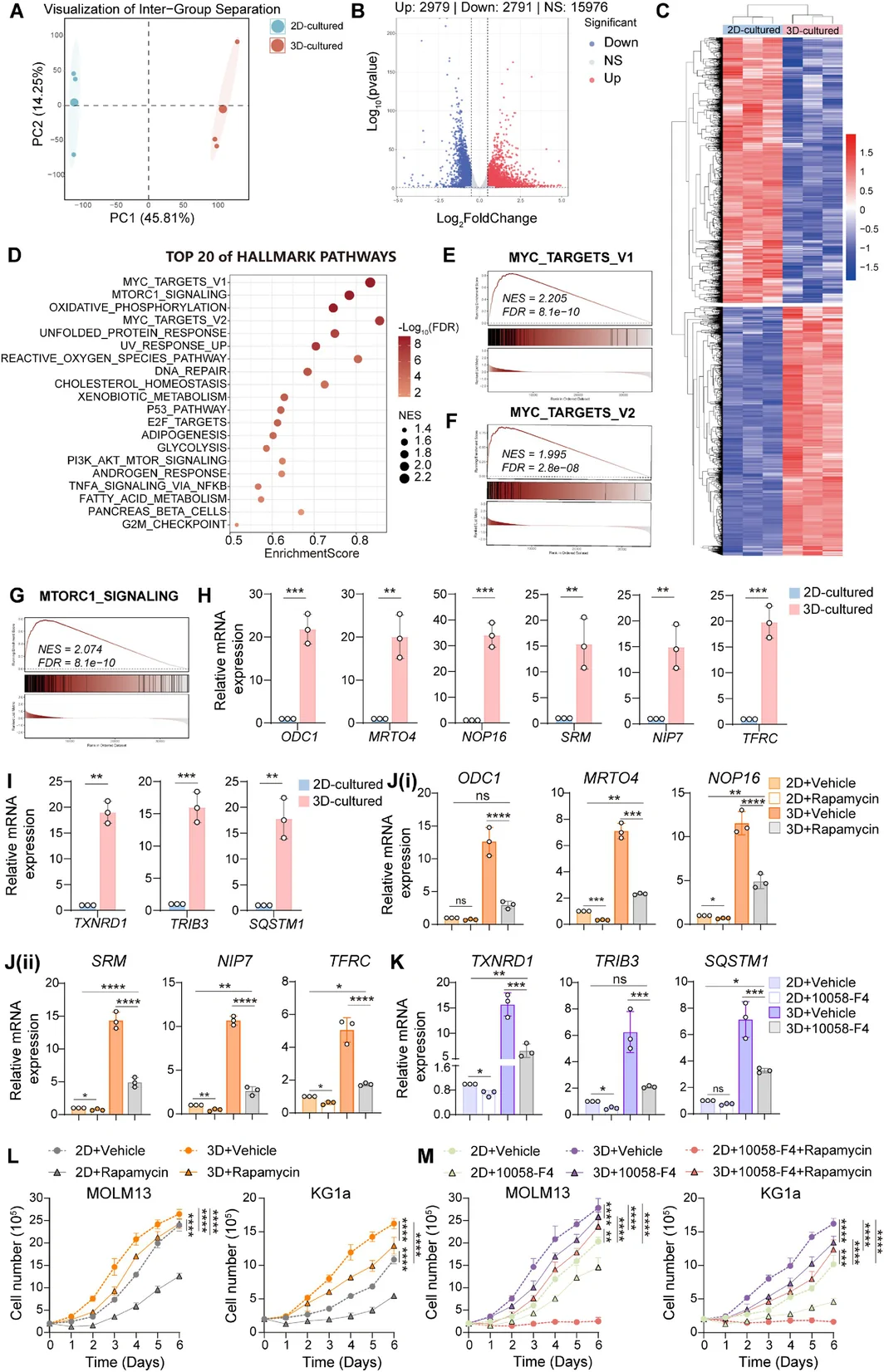
Enhanced AML cell survival and proliferation in 3D culture is associated with activation of MYC and mTORC1 signalling. (A) Principal component analysis based on RNA‐seq expression profiles of BMMNCs cultured in 2D and 3D systems (*n* = 3 technical replicates). (B) Volcano plot comparing 3D‐ versus 2D‐cultured BMMNCs. Significantly altered genes (*p* < 0.05, |log_2_FC| > 0.5) are highlighted (*n* = 3 technical replicates). (C) Heatmap showing significantly altered genes between 3D‐ and 2D‐cultured BMMNCs (*n* = 3 technical replicates). (D) GSEA using Hallmark gene sets showing the top 20 enriched pathways in 3D‐cultured BMMNCs. (E, F) GSEA enrichment plots for MYC target signatures in 3D‐cultured BMMNCs, with NES and FDR values shown. (G) GSEA enrichment plot for the mTORC1 signalling signature in 3D‐cultured BMMNCs, with NES and FDR values shown. (H, I) The mRNA expression levels of key genes of MYC targets and mTORC1 signalling in BMMNCs, as determined by RT‐qPCR (*n* = 3 independent experiments). (J) The mRNA expression levels of key genes of MYC targets in BMMNCs following rapamycin treatment, as determined by RT‐qPCR (*n* = 3 independent experiments). (K) The mRNA expression levels of key genes of mTORC1 signalling in BMMNCs following 10058‐F4 treatment, as determined by RT‐qPCR (*n* = 3 independent experiments). (L) Growth curves of MOLM13 and KG1a cells analysed over 6 days of 2D and 3D culture following rapamycin treatment (*n* = 3 technical replicates). (M) Growth curves of MOLM13 and KG1a cells analysed over 6 days of 2D and 3D culture following treatment with 10058‐F4 alone or in combination with rapamycin (*n* = 3 technical replicates). Data are shown as mean ± SD, by unpaired Student's *t*‐test (H, I), one‐way ANOVA (J, K) or two‐way ANOVA (L, M). (*p < 0.05, **p < 0.01, ***p < 0.001, ****p < 0.0001, ns, not significant). NES, normalised enrichment scores.

We further performed Gene Set Enrichment Analysis (GSEA) using the HALLMARK gene sets. Among the top 20 most significantly enriched pathways, the majority were related to cell proliferation and survival, including MYC targets, mTORC1 signalling, E2F targets, PI3K‐AKT–mTOR signalling, and TNFα signalling via NF‐κB (Figure 3D). Notably, the most significantly enriched pathways included MYC targets and mTORC1 signalling (Figures 3E–G and S2A–C). To confirm these findings at the mRNA level, we performed qPCR on genes within these pathways. Expression of *TFRC*, *TXNRD1*, *TRIB3*, *SRM*, *SQSTM1*, *ODC1*, *NOP16*, *NIP7* and *MRTO4* was significantly upregulated in the 3D‐cultured cells, consistent with the pathway enrichment results (Figure 3H,I). These molecular changes are consistent with and may contribute to the increased cell numbers and enhanced proliferation observed in 3D‐cultured AML cells.

To further dissect the role of MYC targets and mTORC1 signalling in mediating the pro‐survival and pro‐proliferative effects of 3D culture, we performed pharmacological rescue experiments using the c‐MYC inhibitor 10058‐F4 and the mTORC1 inhibitor rapamycin. A previous study has suggested the existence of a positive feedback loop between MYC and mTORC1 signalling in AML [27]. To investigate this mechanism, we included two AML cell lines with distinct genetic backgrounds, MOLM13 and KG1a. RT‐qPCR analysis showed that rapamycin treatment partially attenuated the upregulation of MYC target genes in MOLM13 and KG1a cells under 3D culture conditions (Figure 3J). Conversely, treatment with 10058‐F4 partially reduced the expression of mTORC1 signalling‐associated genes in the 3D group (Figure 3K). These results indicate that 3D culture activates a MYC‐mTORC1 positive feedback loop in AML cells. Functionally, treatment with either 10058‐F4 or rapamycin alone partially rescued the enhanced proliferative phenotype observed in 3D culture, reducing proliferation by approximately 40%–60%. Notably, combined treatment with both inhibitors led to a more pronounced effect, attenuating ~80% of the growth advantage conferred by 3D culture (Figure 3L,M).

Together, these findings suggest that the 3D culture environment promotes a hyperproliferative state in AML cells that is, at least in part, mediated by activation of a MYC‐mTORC1 positive feedback axis.

### 3.4 3D Culture Enhances the Global Metabolic Activity of AML Cells

Interestingly, our transcriptomic analysis also revealed significant enrichment of oxidative phosphorylation (OXPHOS) pathways in AML cells in 3D‐culture (Figures 3D, 4A and S3A). qPCR further confirmed marked upregulation of genes encoding respiratory chain complexes I–V and coenzyme Q biosynthesis in the 3D group, including *NDUFS1*, *NDUFA8*, *SDHA*, *UQCR10*, *COX5A*, *COX7A2*, *ATP5F1A*, *ATP5PB* and *COQ3* (Figure S3B–G). Because OXPHOS relies on carbohydrates, fatty acids, and amino acids as substrates for mitochondrial aerobic respiration, we next examined upstream metabolic pathways. We found that fatty acid metabolism (Figure 4B), pyruvate metabolism (Figure 4C), and amino acid metabolism (Figure 4C) were all upregulated in 3D‐cultured cells, suggesting a global enhancement of aerobic metabolism. To further validate these findings, we evaluated mitochondrial respiration using the Seahorse Cell Mito Stress Test. We included two AML cell lines with distinct genetic backgrounds (MOLM13 and KG1a) to minimise the confounding effects of inter‐patient heterogeneity. Consistent with the transcriptomic signatures, basal respiration, maximal respiration, and ATP production were all markedly elevated in primary BMMNCs (Figure 4E), as well as in MOLM13 and KG1a cells (Figure 4F), following 3D culture relative to 2D conditions. These results indicate that 3D culture robustly enhances mitochondrial respiratory capacity in AML cells.

**FIGURE 4:**
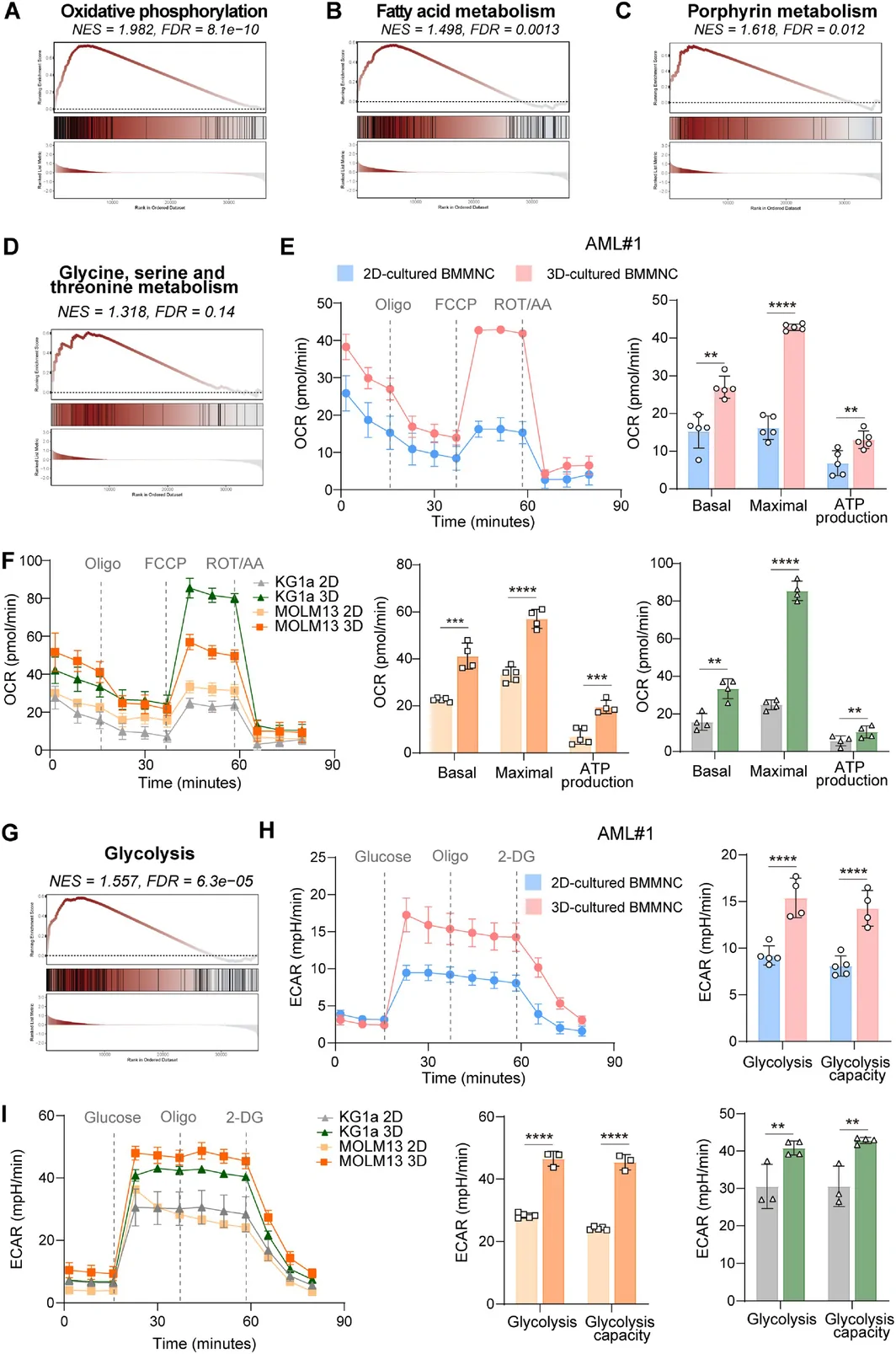
3D culture enhances the global metabolic activity of AML cells. (A) GSEA enrichment plot for the oxidative phosphorylation signature in 3D‐cultured BMMNCs, with normalised enrichment score (NES) and FDR values shown. (B) GSEA enrichment plot for the fatty acid metabolism signature in 3D‐cultured BMMNCs, with NES and FDR values shown. (C) GSEA enrichment plot for the porphyrin metabolism signature in 3D‐cultured BMMNCs, with NES and FDR values shown. (D) GSEA enrichment plot for the Glycine, serine, and threonine metabolism signature in 3D‐cultured BMMNCs, with NES and FDR values shown. (E) OCR measurements of BMMNCs cultured in 2D versus 3D systems (*n* = 5 technical replicates). (F) OCR measurements of MOLM13 and KG1a cells cultured in 2D versus 3D systems (*n* = 4–5 technical replicates). (G) GSEA enrichment plot for the glycolysis signature in 3D‐cultured BMMNCs, with NES and FDR values shown. (H) ECAR measurements of BMMNCs cultured in 2D versus 3D systems (*n* = 4–5 technical replicates). (I) ECAR measurements of MOLM13 and KG1a cells cultured in 2D versus 3D systems (*n* = 3–5 technical replicates). Data are shown as mean ± SD, by unpaired Student's *t*‐test (E–I) (*p < 0.05, **p < 0.01, ***p < 0.001, ****p < 0.0001, ns, not significant). 2‐DG, 2‐deoxy‐glucose; ECAR, extracellular acidification rate; OCR, oxygen consumption rate; Oligo, oligomycin.

In addition to mitochondrial respiration, glycolysis represents another major pathway for ATP generation and is widely reported to be a predominant metabolic mode in tumour cells, including AML. Notably, glycolysis was one of the most significantly enriched pathways, ranking within the top 20 in the GSEA (Figures 3D, 4G and S4A). qPCR confirmed elevated expression of several rate‐limiting glycolytic enzymes, including *PFKP*, *HK1*, and *PKM*, as well as *PGK1*, a key enzyme associated with tumour glycolytic activity (Figure S4B–F). We further evaluated glycolytic function using the Seahorse Glycolysis Stress Test. Primary BMMNCs, Molm13, and KG1a cells all exhibited significantly increased glycolysis and glycolytic capacity after 3D culture relative to 2D conditions (Figure 4H,I). These findings indicate that 3D culture substantially boosts glycolytic activity in AML cells.

Together, these data indicate that 3D‐cultured AML cells exhibit broadly upregulated metabolic activity, potentially underpinning their increased proliferative potential.

### 3.5 3D Bioprinting Recapitulates Clinically Relevant Drug Resistance Phenotypes

To further evaluate the drug sensitivity of the 3D bioprinted AML drug screening platform, 2D‐ and 3D‐cultured BMMNCs were treated with Cytarabine (AraC) and Daunorubicin (DNR), which are the standard chemotherapeutic agents for AML. IC_50_ concentrations were measured at 24 h and 72 h post‐treatment, revealing that AML cells cultured in 3D exhibited markedly increased drug resistance. Specifically, after 72 h treatment, for AML#1, the IC_50_ for AraC was 4.12 μM in 2D cultures and 65.16 μM in 3D cultures, while DNR showed IC_50_ values of 3.14 and 6.76 μM, respectively (Figure 5A,B). These IC_50_ values were subsequently used to define the ‘low’ and ‘high’ drug concentrations for each group in downstream experiments. Notably, similar trends were observed in two additional AML patient samples (AML#2 and AML#3), further supporting the robustness of this finding (Figure S5A–B).

**FIGURE 5:**
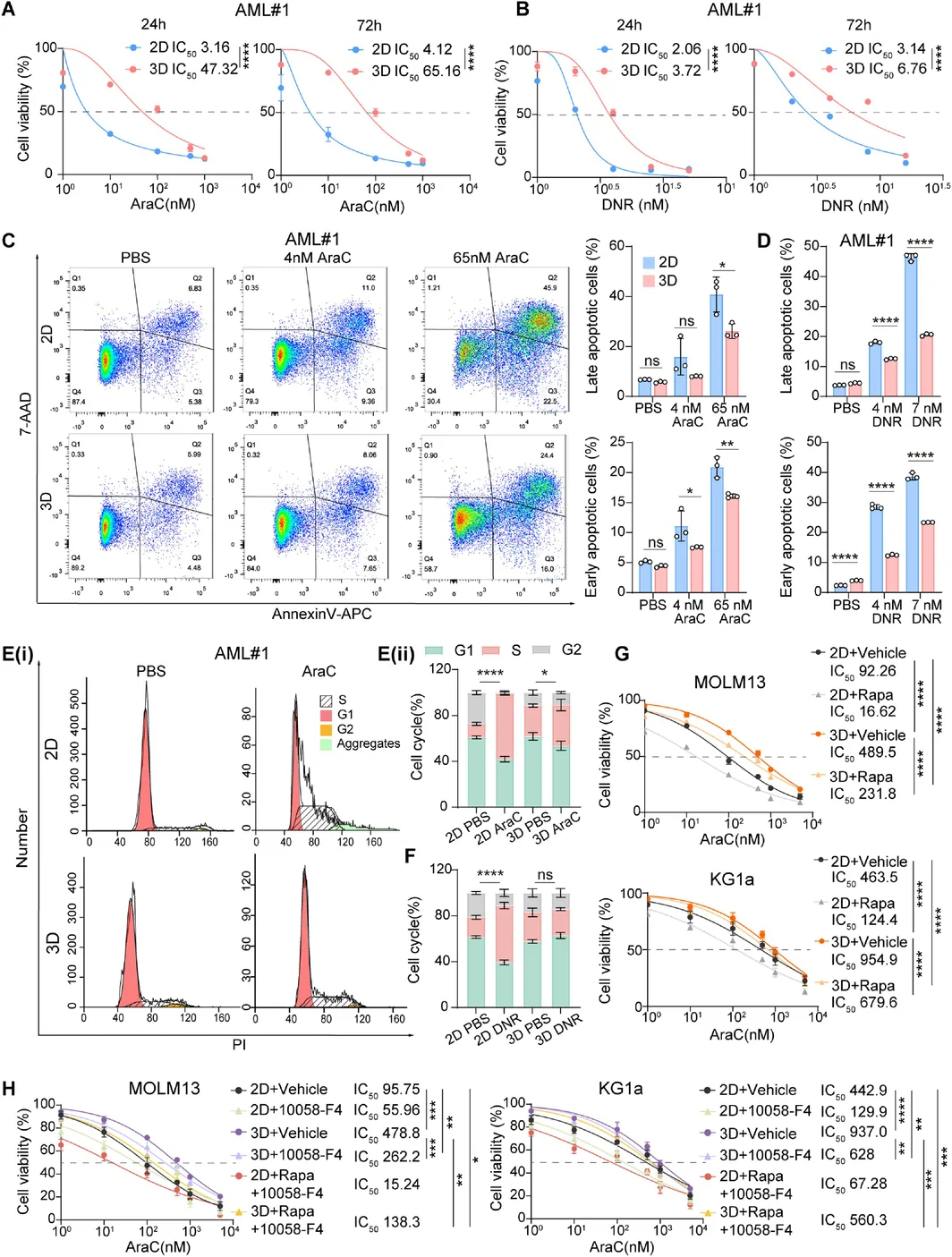
3D bioprinting recapitulates the drug‐tolerant behaviour of leukaemia cells. (A) Dose–response curves and IC_50_ of 3D‐cultured BMMNCs treated with Ara‐C for 24 and 72 h (*n* = 3 technical replicates). (B) Dose–response curves and IC_50_ of 3D‐cultured BMMNCs treated with DNR for 24 and 72 h (*n* = 3 technical replicates). (C) BMMNCs cultured in 2D or 3D systems were treated with 4 or 65 nM Ara‐C for 72 h. Representative FACS plots showing Annexin V^+^7‐AAD^−^ (early apoptosis) and Annexin V^+^7‐AAD^+^ (late apoptosis) populations, along with their quantified frequencies (*n* = 3 technical replicates). (D) BMMNCs cultured in 2D or 3D systems were treated with 3 or 7 nM DNR for 72 h. The percentages of early and late apoptotic cells were determined by FACS (*n* = 3 technical replicates). (E) BMMNCs cultured in 2D or 3D systems were treated with 65 nM Ara‐C for 24 h. Representative PI histograms show cell‐cycle distributions based on DNA content, along with their quantified frequencies (*n* = 3 technical replicates). (F) BMMNCs cultured in 2D or 3D systems were treated with 7 nM DNR for 24 h, and cell‐cycle profiles were analysed by flow cytometry (*n* = 3 technical replicates). (G) Dose–response curves and IC_50_ of 3D‐cultured MOLM13 and KG1a cells treated with AraC alone or in combination with rapamycin for 24 h (*n* = 3 technical replicates). (H) Dose–response curves and IC_50_ of 3D‐cultured MOLM13 and KG1a cells treated with AraC,10058‐F4 and rapamycin for 24 h (*n* = 3 technical replicates). Data are shown as mean ± SD, by unpaired Student's *t*‐test (A, B) or one‐way ANOVA (C–H) (*p < 0.05, **p < 0.01, ***p < 0.001, ****p < 0.0001, ns, not significant). AraC, Cytarabine; DNR, Daunorubicin; Rapa, rapamycin.

Flow cytometry analysis of apoptosis revealed that, after 72 h of AraC treatment, both early and late apoptotic fractions were significantly lower in the 3D group compared with 2D (Figures 5C and S5C). Likewise, following treatment with DNR, 3D‐cultured cells also exhibited markedly reduced levels of early and late apoptosis relative to the 2D controls (Figure 5D and S5D), indicating that AML cells cultured in 3D are more resistant to chemotherapeutic agents.

Cell cycle analysis revealed that 24 h treatment with high‐dose AraC or DNR led to a significant increase in S‐phase cells in 2D culture, whereas 3D‐cultured cells showed only a modest or no significant change, suggesting that drug‐induced cell cycle arrest is attenuated in 3D (Figures 5E,F and S5E,F). Together, these findings demonstrate that AML cells in 3D culture display enhanced chemoresistance, more closely recapitulating the in vivo behaviour of leukaemia cells.

Next, we sought to determine whether the increased chemoresistance observed in 3D‐cultured AML cells is associated with activation of the MYC‐mTORC1 positive feedback loop, as both pathways have been previously implicated in drug resistance in AML [28, 29, 30]. Following 24 h of treatment with either the c‐MYC inhibitor 10058‐F4 or the mTORC1 inhibitor rapamycin in combination with AraC, the IC_50_ values of MOLM13 and KG1a cells cultured in 3D were partially reduced. Notably, combined inhibition of MYC and mTORC1 resulted in a more pronounced effect, decreasing the IC_50_ in the 3D group by over 80% (Figure 5G,H). These findings indicate that activation of the MYC‐mTORC1 positive feedback loop plays a key role in mediating the chemo‐resistant phenotype of AML cells under 3D culture conditions.

## 4 Discussion

In this study, we establish a reproducible 3D bioprinted bone‐marrow‐like platform generated from patient‐derived AML BMMNCs and a mechanically tunable GelHA bioink. Compared with conventional 2D culture, our patient‐derived 3D‐bioprinted GelHA platform sustained primary AML cells with significantly enhanced viability, proliferation, stem cell phenotype retention, metabolic activity, and chemoresistance to agents like AraC and DNR. Mechanistically, the MYC‐mTORC1 axis plays a pivotal role in sustaining these phenotypes within the 3D bioprinted microenvironment. Taken together, these findings establish that the bioprinted microenvironment faithfully recapitulated in vivo disease biology, offering a platform of substantial value for mechanistic and translational discovery.

Recent advances in 3D bone marrow modelling highlight that the ideal disease model should combine high physiological fidelity with scalability for drug screening [31]. Although existing 3D systems have improved in vitro relevance, key limitations remain in cell source, structural control and culture complexity. Most studies rely on immortalised AML cell lines such as HL‐60, THP‐1 and K562 [14, 18], which may progressively lose critical pathological characteristics during prolonged culture. In contrast, we use patient‐derived primary cells throughout, preserving genetic heterogeneity and enabling more clinically relevant readouts. Notably, primary AML blasts remain viable through bioprinting and photocrosslinking, retaining their phenotype and functional responsiveness. With regard to structural control, a range of 3D platforms, including spheroids, hydrogel‐based cultures, scaffolds, and organ‐on‐chip systems [7, 32], have been developed, but generally offer limited spatial precision. Self‐organising organoid models [33] introduce additional variability in size, structure, and maturation, thereby limiting reproducibility. By contrast, bioprinting enables precise control over architecture and transport properties, while tuning GelHA concentration allows matrix stiffness to approximate native bone marrow. These features likely contribute to the maintenance of stem‐like phenotypes, enhanced proliferative capacity, and metabolic activity. In addition, many systems rely on exogenous cytokines or complex induction strategies [34], increasing cost and limiting scalability. Our platform maintains high primary cell viability without exogenous factors, reducing complexity and improving translational potential. Overall, the combination of patient‐derived cells and a reproducible architecture compatible with high‐throughput production positions our 3D bioprinted platform as a promising tool for ex vivo drug screening. Consistent with previous studies [35, 36, 37, 38], we find that 3D culture supports AML cell survival and drug resistance, which may reduce false‐positive leads in preclinical studies and improve the prioritisation of candidate therapies. In the context of personalised medicine, limited amounts of diagnostic or pre‐treatment bone marrow can be used to rapidly generate multiple assayable constructs, enabling individualised drug sensitivity testing prior to therapy selection.

Although our 3D printing workflow preserves key AML phenotypes, the current constructs primarily capture mechanical and topological cues and do not include non‐haematopoietic niche components (e.g., mesenchymal stromal cells, endothelial cells and osteoblasts) [23]. This simplification enables us to isolate the effects of matrix mechanics and spatial architecture while minimising confounding signals, and also improves clinical applicability where matched niche cells are often unavailable [23, 39]. However, it limits the ability to model direct cell–cell interactions between niche cells and leukaemic blasts. Future work will focus on incorporating key microenvironmental populations, such as mesenchymal stromal cells, and exploring co‐printing strategies to generate vascularized bone marrow organoids. Nonetheless, building multicellular bone marrow models remains challenging, as different cell types require distinct culture conditions, physiologically relevant cell ratios are difficult to achieve, and rare populations such as MSCs (~0.001%–0.01% of BMMNCs) [40] are difficult to maintain ex vivo. In addition, the spatial heterogeneity of the bone marrow niche further complicates faithful reconstruction using current bioprinting approaches.

Mechanistically, we find that the enhanced proliferative capacity observed in the 3D‐bioprinted AML model is primarily driven by a MYC‐mTORC1 feed‐forward regulatory circuit. The 3D architecture likely establishes physiologically relevant nutrient and oxygen gradients, thereby activating mTORC1 as a central nutrient sensor. Activated mTORC1, in turn, reinforces MYC activity at multiple levels, while MYC, as a global transcriptional amplifier of metabolic programs, further potentiates mTORC1 signalling, forming a self‐sustaining positive feedback loop. This circuit drives coordinated metabolic rewiring in AML cells: MYC directly induces key glycolytic genes (e.g., HK2 and LDHA) [41, 42, 43, 44], as well as anabolic pathways including serine‐glycine metabolism [45] and nucleotide biosynthesis [46], and promotes mitochondrial biogenesis and oxidative phosphorylation [41, 47, 48]. Together, these processes coordinate bioenergetic and biosynthetic outputs to support sustained proliferation and metabolic fitness in the 3D context.

In parallel, activation of the MYC‐mTORC1 axis provides a mechanistic basis for the chemo‐resistant phenotype induced by 3D culture. Our data indicate that this pathway is a central driver of drug resistance in AML cells under 3D conditions. Consistent with previous studies, MYC can promote therapy resistance through multiple mechanisms [30, 49, 50, 51], including metabolic reprogramming, enhanced DNA damage repair, suppression of apoptosis, and maintenance of cancer stem cell properties. These features are in line with our observations of increased metabolic activity and stem‐like characteristics in 3D‐cultured AML cells.

In summary, we present a 3D‐bioprinted platform that transforms primary AML samples into a clinically predictive ex vivo model. This system not only preserves disease biology but also actively drives its hallmark aggressive phenotypes: proliferation, metabolic activation and chemoresistance. By bridging the critical gap between simplistic 2D cultures and the in vivo marrow microenvironment, our approach offers a scalable, patient‐specific pathway to revolutionise preclinical drug testing and accelerate personalised therapy development in leukaemia.

## Author Contributions

W.H.: methodology, investigation, formal analysis and writing – original draft. B.D.: methodology, visualisation and investigation. N.G.: investigation, formal analysis and validation. Y.D.: validation. Z.X.: validation. Y.L.: validation. Y.M.: resources. Q.R.: resources. N.W.: formal analysis, writing – review and editing. P.H.: conceptualisation, funding acquisition and writing – review and editing. X.M.: conceptualisation, supervision, funding acquisition and writing – review and editing.

## Funding

This work was supported by the National Key R&D Program of China, 2021YFA1100903; CAMS Innovation Fund for Medical Sciences, 2023‐I2M‐2‐007, 2021‐I2M‐1‐019; the Fundamental Research Funds for Central Universities, Peking Union Medical College, 3332025143; National Natural Science Foundation of China, 82270122, 82570157; National Key R&D Program of China, 2024YFA1107700; CAMS Innovation Fund for Medical Sciences, 2021‐I2M‐1‐058; The Non‐profit Central Research Institute Fund of Chinese Academy of Medical Sciences, 2023‐PT310‐05.

## Ethics Statement

All procedures followed were in accordance with the ethical standards of the responsible committee on human experimentation (institutional and national) and with the Helsinki Declaration of 1975, as revised in 2008. Informed consent was obtained from all patients for being included in the study.

## Conflicts of Interest

The authors declare no conflicts of interest.

## Supporting information


**Figure S1:** Characterisation of AML cells in 3D culture conditions. (A) Representative fluorescence‐activated cell sorting (FACS) plots showing CD45⁺CD34⁺CD38⁻ LSCs in BMMNCs from the 2D and 3D culture at day 0 and day 6. (B) Representative Sanger sequencing chromatograms showing AML‐associated hotspot mutations (arrowheads) in genomic DNA extracted from BMMNCs of AML#3 after 6 days of 2D or 3D culture. (C) Bright‐field microscopy images of the printed structures. (D) Representative live/dead staining images and assessment of cell viability of BMMNCs‐laden constructs at days 0, 2, 4, 6 and 8 post‐printing (*n* = 5 technical replicates). Live and dead cells were labelled with Calcein‐AM (green) and PI (red), respectively. Scale bar, 200 μm. Data are shown as mean ± SD, by one‐way ANOVA (D) (**p < 0.01, ns, not significant). LSC, leukaemic stem cell.
**Figure S2:** Heatmaps of MYC target enrichment (A, B) and mTORC1 signalling enrichment (C) based on RNA‐seq data from 3D‐cultured BMMNCs.
**Figure S3:** 3D culture enhances OXPHOS of AML cells. (A) Heatmaps of oxidative phosphorylation enrichment based on RNA‐seq data from 3D‐cultured BMMNCs. (B–G) The mRNA expression levels of respiratory chain complexes I–V and coenzyme Q in BMMNCs of AML#1, as determined by RT‐ qPCR (*n* = 3 independent experiments). Data are shown as mean ± SD, by Student's t‐test (*p < 0.05, **p < 0.01, ***p < 0.001).
**Figure S4:** 3D culture enhances glycolysis of AML cells. (A) Heatmaps of glycolysis enrichment based on RNA‐seq data from 3D‐cultured BMMNCs. (B) Schematic diagram of glycolysis. (C–F) The mRNA expression levels of glycolytic enzymes in BMMNC of AML#1, as determined by RT‐qPCR (*n* = 3 independent experiments). Data are shown as mean ± SD, by Student's t‐test (*p < 0.05, **p < 0.01, ***p < 0.001).
**Figure S5:** 3D bioprinting recapitulates drug tolerance in leukaemia cells. (A) Dose–response curves and IC50 of 3D‐cultured BMMNCs treated with AraC for 24 h (*n* = 3 technical replicates). (B) Dose–response curves and IC50 of 3D‐cultured BMMNCs treated with DNR for 24 h (*n* = 3 technical replicates). (C) BMMNCs cultured in 2D or 3D systems were treated with AraC for 72 h. The percentages of early and late apoptotic cells were determined by flow cytometry (*n* = 3 technical replicates). (D) BMMNCs cultured in 2D or 3D systems were treated with DNR for 72 h. The percentages of early and late apoptotic cells were determined by flow cytometry (*n* = 3 technical replicates). (E) BMMNCs cultured in 2D or 3D systems were treated with AraC (AML#2225 nM, AML#3140 nM) for 24 h, and cell‐cycle profiles were analysed by flow cytometry (*n* = 3 technical replicates). (F) BMMNCs cultured in 2D or 3D systems were treated with DNR (AML#2 5 nM, AML#3 6 nM) for 24 h, and cell‐cycle profiles were analysed by flow cytometry (*n* = 3 technical replicates). Data are shown as mean ± SD, by unpaired Student's t‐test (A, B) or one‐way ANOVA (C–F) (*p < 0.05, **p < 0.01, ***p < 0.001, ****p < 0.0001, ns, not significant). AraC, Cytarabine; DNR, Daunorubicin.
**Table S1:** Sequences of forward and reverse primers used in PCR amplification.
**Table S2:** Sequences of forward and reverse primers used in RT‐qPCR assays.

## Data Availability

Raw RNA sequencing data have been deposited in the Genome Sequence Archive (GSA) for Human at the National Genomics Data Center, under the BioProject accession number HRA018188.

## References

[1] C. D. DiNardo , H. P. Erba , S. D. Freeman , and A. H. Wei , “Acute Myeloid Leukaemia,” Lancet 401, no. 10393 (2023): 2073–2086, 10.1016/s0140-6736. 37068505

[2] R. S. Bhansali , K. W. Pratz , and C. Lai , “Recent Advances in Targeted Therapies in Acute Myeloid Leukemia,” Journal of Hematology and Oncology 16, no. 1 (2023): 29, 10.1186/s13045-023-01424-6. PMC10039574 36966300

[3] S. Vasu , J. Kohlschmidt , K. Mrózek , et al., “Ten‐Year Outcome of Patients With Acute Myeloid Leukemia Not Treated With Allogeneic Transplantation in First Complete Remission,” Blood Advances 2, no. 13 (2018): 1645–1650, 10.1182/bloodadvances.2017015222. PMC6039651 29991495

[4] M. S. Tallman , D. G. Gilliland , and J. M. Rowe , “Drug Therapy for Acute Myeloid Leukemia,” Blood 106, no. 4 (2005): 1154–1163, 10.1182/blood-2005-01-0178. 15870183

[5] X. S. Zhao , X. T. Chen , and Y. J. Chang , “Stem Cell Transplantation Indications for Patients With Acute Leukemia Determined by Measurable Residual Disease: What We Know and What We Do Not Know,” Blood Science 7 (2025): e00229, 10.1097/bs9.0000000000000229. PMC11939945 40144893

[6] F. Pampaloni , E. G. Reynaud , and E. H. K. Stelzer , “The Third Dimension Bridges the Gap Between Cell Culture and Live Tissue,” Nature Reviews Molecular Cell Biology 8, no. 10 (2007): 839–845, 10.1038/nrm2236. 17684528

[7] G. Silvestri and A. Chatterjee , “Rebuilding the Marrow In Vitro: Translational Advances in the 3D Modeling of Blood Cancers,” Oncologist 5 (2025): 51, 10.3390/onco5040051. PMC12674608 41346447

[8] G. Egan and A. D. Schimmer , “Contribution of Metabolic Abnormalities to Acute Myeloid Leukemia Pathogenesis,” Trends in Cell Biology 33, no. 6 (2023): 455–462, 10.1016/j.tcb.2022.11.004. 36481232

[9] J. K. Lee , Z. Liu , J. K. Sa , et al., “Pharmacogenomic Landscape of Patient‐Derived Tumor Cells Informs Precision Oncology Therapy,” Nature Genetics 50, no. 10 (2018): 1399–1411, 10.1038/s41588-018-0209-6. PMC8514738 30262818

[10] X. Zhang , D. Cao , L. Xu , et al., “Harnessing Matrix Stiffness to Engineer a Bone Marrow Niche for Hematopoietic Stem Cell Rejuvenation,” Cell Stem Cell 30, no. 4 (2023): 378–395.e8, 10.1016/j.stem.2023.03.005. 37028404

[11] D. Tuveson and H. Clevers , “Cancer Modeling Meets Human Organoid Technology,” Science 364, no. 6444 (2019): 952–955, 10.1126/science.aaw6985. 31171691

[12] X. Y. Tang , S. Wu , D. Wang , et al., “Human Organoids in Basic Research and Clinical Applications,” Signal Transduction and Targeted Therapy 7, no. 1 (2022): 168, 10.1038/s41392-022-01024-9. PMC9127490 35610212

[13] N. B. Robinson , K. Krieger , F. M. Khan , et al., “The Current State of Animal Models in Research: A Review,” International Journal of Surgery 72 (2019): 9–13, 10.1016/j.ijsu.2019.10.015. 31627013

[14] A. Sharipol and B. J. Frisch , “Are We Ready to Integrate 3D Culture Systems in Acute Myeloid Leukemia and Bone Marrow Microenvironment Research?,” Frontiers in Hematology 3 (2024): 1407698, 10.3389/frhem.2024.1407698.

[15] P. Shukla , S. Yeleswarapu , M. A. Heinrich , J. Prakash , and F. Pati , “Mimicking Tumor Microenvironment by 3D Bioprinting: 3D Cancer Modeling,” Biofabrication 14, no. 3 (2022): 032002, 10.1088/1758-5090/ac6d11. 35512666

[16] G. Decante , J. B. Costa , J. Silva‐Correia , et al., “Engineering Bioinks for 3D Bioprinting,” Biofabrication 13 (2021): 032001, 10.1088/1758-5090/abec2c. 33662949

[17] D. Ashok , L. Polcik , S. Dannewitz Prosseda , et al., “Insights Into Bone Marrow Niche Stability: An Adhesion and Metabolism Route,” Frontiers in Cell and Developmental Biology 9 (2021): 798604, 10.3389/fcell.2021.798604. PMC8806031 35118078

[18] D. M. Alhattab , I. Isaioglou , S. Alshehri , et al., “Fabrication of a Three‐Dimensional Bone Marrow Niche‐Like Acute Myeloid Leukemia Disease Model by an Automated and Controlled Process Using a Robotic Multicellular Bioprinting System,” Biomaterials Research 27, no. 1 (2023): 111, 10.1186/s40824-023-00457-9. PMC10626721 37932837

[19] H. Zhou , M. Wu , Z. Ding , et al., “A Hyaluronic Acid‐Enhanced 3D‐Bioprinted Osteosarcoma Model Reveals Mechanisms of Tumor Metastasis and Chemoresistance,” Bio‐Design and Manufacturing 8, no. 5 (2025): 724–741, 10.1631/bdm.2400390.

[20] F. V. Sbrana , R. Pinos , F. Barbaglio , et al., “3D Bioprinting Allows the Establishment of Long‐Term 3D Culture Model for Chronic Lymphocytic Leukemia Cells,” Frontiers in Immunology 12 (2021): 639572, 10.3389/fimmu.2021.639572. PMC8126722 34012434

[21] “FDA Pushes to Replace Animal Testing,” Nature Biotechnology 43, no. 5 (2025): 655, 10.1038/s41587-025-02690-0. 40380010

[22] S. L. Ellis , J. Grassinger , A. Jones , et al., “The Relationship Between Bone, Hemopoietic Stem Cells, and Vasculature,” Blood 118, no. 6 (2011): 1516–1524, 10.1182/blood-2010-08-303800. 21673348

[23] S. Pinho and P. S. Frenette , “Haematopoietic Stem Cell Activity and Interactions With the Niche,” Nature Reviews. Molecular Cell Biology 20, no. 5 (2019): 303–320, 10.1038/s41580-019-0103-9. PMC6483843 30745579

[24] P. A. Dinndorf , R. G. Andrews , D. Benjamin , D. Ridgway , L. Wolff , and I. D. Bernstein , “Expression of Normal Myeloid‐Associated Antigens by Acute Leukemia Cells,” Blood 67, no. 4 (1986): 1048–1053. 2937468

[25] K. J. Hope , L. Jin , and J. E. Dick , “Acute Myeloid Leukemia Originates From a Hierarchy of Leukemic Stem Cell Classes That Differ in Self‐Renewal Capacity,” Nature Immunology 5, no. 7 (2004): 738–743, 10.1038/ni1080. 15170211

[26] D. Bonnet and J. E. Dick , “Human Acute Myeloid Leukemia Is Organized as a Hierarchy That Originates From a Primitive Hematopoietic Cell,” Nature Medicine 3, no. 7 (1997): 730–737, 10.1038/nm0797-730. 9212098

[27] A. G. Davis , D. T. Johnson , D. Zheng , et al., “Alternative Polyadenylation Dysregulation Contributes to the Differentiation Block of Acute Myeloid Leukemia,” Blood 139, no. 3 (2022): 424–438, 10.1182/blood.2020005693. PMC8777198 34482400

[28] T. Hoshii , Y. Tadokoro , K. Naka , et al., “mTORC1 Is Essential for Leukemia Propagation but Not Stem Cell Self‐Renewal,” Journal of Clinical Investigation 122, no. 6 (2012): 2114–2129, 10.1172/jci62279. PMC3366413 22622041

[29] B. Xia , C. Tian , S. Guo , et al., “C‐Myc Plays Part in Drug Resistance Mediated by Bone Marrow Stromal Cells in Acute Myeloid Leukemia,” Leukemia Research 39, no. 1 (2015): 92–99, 10.1016/j.leukres.2014.11.004. 25443862

[30] C. Fauriat and D. Olive , “AML Drug Resistance: C‐Myc Comes Into Play,” Blood 123, no. 23 (2014): 3528–3530, 10.1182/blood-2014-04-566711. 24904096

[31] P. Scala , B. Serio , and V. Giudice , “3D In Vitro Models of the Bone Marrow Niche,” ACS Biomaterials Science and Engineering 12, no. 1 (2026): 110–127, 10.1021/acsbiomaterials.5c01421. PMC12801197 41384609

[32] J. Lv , X. Du , M. Wang , et al., “Construction of Tumor Organoids and Their Application to Cancer Research and Therapy,” Theranostics 14 (2024): 1101–1125, 10.7150/thno.91362. PMC10797287 38250041

[33] M. Giannini , G. Campione , L. Torcq , et al., “A Human Pathophysiological 3D‐Bone Marrow Model Reveals Immune and Stromal Cell Heterogeneity,” Communications Biology 9, no. 1 (2026): 164, 10.1038/s42003-025-09433-6. PMC12873127 41484482

[34] K. Ren , E. Li , I. Aydemir , et al., “Development of iPSC‐Derived Human Bone Marrow Organoid for Autonomous Hematopoiesis and Patient‐Derived HSPC Engraftment,” Blood Advances 9, no. 1 (2025): 54–65, 10.1182/bloodadvances.2024013361. PMC11732577 39471483

[35] H. L. Cheung , Y. H. Wong , Y. Y. Li , et al., “Microenvironment Matters: In Vitro 3D Bone Marrow Niches Differentially Modulate Survival, Phenotype and Drug Responses of Acute Myeloid Leukemia (AML) Cells,” Biomaterials 312 (2025): 122719, 10.1016/j.biomaterials.2024.122719. 39088912

[36] M. B. Dainiak , I. N. Savina , I. Musolino , A. Kumar , B. Mattiasson , and I. Y. Galaev , “Biomimetic Macroporous Hydrogel Scaffolds in a High‐Throughput Screening Format for Cell‐Based Assays,” Biotechnology Progress 24, no. 6 (2008): 1373–1383, 10.1002/btpr.30. 19194952

[37] M. S. Nair , U. Mony , D. Menon , et al., “Development and Molecular Characterization of Polymeric Micro‐Nanofibrous Scaffold of a Defined 3‐D Niche for In Vitro Chemosensitivity Analysis Against Acute Myeloid Leukemia Cells,” International Journal of Nanomedicine 10 (2015): 3603–3622, 10.2147/ijn.S80397. PMC4440427 26028971

[38] J. W. Shin and D. J. Mooney , “Extracellular Matrix Stiffness Causes Systematic Variations in Proliferation and Chemosensitivity in Myeloid Leukemias,” Proceedings of The National Academy of Sciences of The United States of America 113, no. 43 (2016): 12126–12131, 10.1073/pnas.1611338113. PMC5086998 27790998

[39] S. Méndez‐Ferrer , D. Bonnet , D. P. Steensma , et al., “Bone Marrow Niches in Haematological Malignancies,” Nature Reviews. Cancer 20, no. 5 (2020): 285–298, 10.1038/s41568-020-0245-2. PMC9912977 32112045

[40] M. F. Pittenger , A. M. Mackay , S. C. Beck , et al., “Multilineage Potential of Adult Human Mesenchymal Stem Cells,” Science 284, no. 5411 (1999): 143–147, 10.1126/science.284.5411.143. 10102814

[41] Z. E. Stine , Z. E. Walton , B. J. Altman , A. L. Hsieh , and C. V. Dang , “MYC, Metabolism, and Cancer,” Cancer Discovery 5, no. 10 (2015): 1024–1039, 10.1158/2159-8290.Cd-15-0507. PMC4592441 26382145

[42] K. C. Patra , Q. Wang , P. T. Bhaskar , et al., “Hexokinase 2 Is Required for Tumor Initiation and Maintenance and Its Systemic Deletion Is Therapeutic in Mouse Models of Cancer,” Cancer Cell 24, no. 2 (2013): 213–228, 10.1016/j.ccr.2013.06.014. PMC3753022 23911236

[43] V. R. Fantin , J. St‐Pierre , and P. Leder , “Attenuation of LDH‐A Expression Uncovers a Link Between Glycolysis, Mitochondrial Physiology, and Tumor Maintenance,” Cancer Cell 9, no. 6 (2006): 425–434, 10.1016/j.ccr.2006.04.023. 16766262

[44] P. Seth , A. Grant , J. Tang , et al., “On‐Target Inhibition of Tumor Fermentative Glycolysis as Visualized by Hyperpolarized Pyruvate,” Neoplasia 13, no. 1 (2011): 60–71, 10.1593/neo.101020. PMC3022429 21245941

[45] V. Cianfanelli , C. Fuoco , M. Lorente , et al., “AMBRA1 Links Autophagy to Cell Proliferation and Tumorigenesis by Promoting c‐Myc Dephosphorylation and Degradation,” Nature Cell Biology 17, no. 1 (2015): 20–30, 10.1038/ncb3072. PMC4976803 25438055

[46] J. van Riggelen , A. Yetil , and D. W. Felsher , “MYC as a Regulator of Ribosome Biogenesis and Protein Synthesis,” Nature Reviews. Cancer 10, no. 4 (2010): 301–309, 10.1038/nrc2819. 20332779

[47] K. S. Jensen , T. Binderup , K. T. Jensen , et al., “FoxO3A Promotes Metabolic Adaptation to Hypoxia by Antagonizing Myc Function,” EMBO Journal 30, no. 22 (2011): 4554–4570, 10.1038/emboj.2011.323. PMC3243591 21915097

[48] H. Zhang , P. Gao , R. Fukuda , et al., “HIF‐1 Inhibits Mitochondrial Biogenesis and Cellular Respiration in VHL‐Deficient Renal Cell Carcinoma by Repression of C‐MYC Activity,” Cancer Cell 11, no. 5 (2007): 407–420, 10.1016/j.ccr.2007.04.001. 17482131

[49] J. Bhin , J. Yemelyanenko , X. Chao , et al., “MYC Is a Clinically Significant Driver of mTOR Inhibitor Resistance in Breast Cancer,” Journal of Experimental Medicine 220 (2023): e20211743, 10.1084/jem.20211743. PMC10465700 37642941

[50] G. Donati and B. Amati , “MYC and Therapy Resistance in Cancer: Risks and Opportunities,” Molecular Oncology 16, no. 21 (2022): 3828–3854, 10.1002/1878-0261.13319. PMC9627787 36214609

[51] X. N. Pan , J. J. Chen , L. X. Wang , et al., “Inhibition of c‐Myc Overcomes Cytotoxic Drug Resistance in Acute Myeloid Leukemia Cells by Promoting Differentiation,” PLoS One 9, no. 8 (2014): e105381, 10.1371/journal.pone.0105381. PMC4134294 25127121

