# Supplementary material for: A 3D Bioprinted Platform That Maintains the Functional Integrity of Primary AML Cells
Source: Cell Prolif. 2026 Jun 16;59(11):e70250. doi: 10.1111/cpr.70250 (PMC13325942; doi:10.1111/cpr.70250)
Supplement: Supplementary file 1 [file CPR-59-e70250-s001.docx]

**Supplementary files**

Supplementary Figures 1-5

Supplementary Tables 1-2

**Supplementary Figure**

**Sup Figure 1.** **Characterization of AML cells in 3D culture conditions**

**(A)** Representative fluorescence-activated cell sorting (FACS) plots showing CD45⁺CD34⁺CD38⁻ LSCs in BMMNCs from the 2D and 3D culture at day 0 and day 6.

**(B)** Representative Sanger sequencing chromatograms showing AML-associated hotspot mutations (arrowheads) in genomic DNA extracted from BMMNCs of AML#3 after 6 days of 2D or 3D culture.

**(C)** Bright-field microscopy images of the printed structures.

**(D)**Representative live/dead staining images and assessment of cell viability of BMMNCs-laden constructs at days 0, 2, 4, 6, and 8 post-printing (n = 5 technical replicates). Live and dead cells were labelled with Calcein-AM (green) and PI (red), respectively. Scale bar, 200 μm.
Data are shown as mean ± SD, by one-way ANOVA (D) (***P* < 0.01, ns, not significant).

LSC, leukemic stem cell.

**Sup Figure 2.** Heatmaps of MYC target enrichment (A-B) and mTORC1 signaling enrichment (C) based on RNA-seq data from 3D-cultured BMMNCs.

**Sup Figure 3. 3D Culture Enhances** **OXPHOS of AML Cells**

**(A)** Heatmaps of oxidative phosphorylation enrichment based on RNA-seq data from 3D-cultured BMMNCs

**(B-G)** The mRNA expression levels of respiratory chain complexes I-V and coenzyme Q in BMMNCs of AML#1, as determined by RT- qPCR (n = 3 independent experiments).

Data are shown as mean ± SD, by Student's t-test (**P* <0.05, ***P* < 0.01, ****P* < 0.001).

**Sup Figure 4. 3D Culture Enhances glycolysis of AML Cells**

**(A)** Heatmaps of glycolysis enrichment based on RNA-seq data from 3D-cultured BMMNCs(

**(B)** Schematic diagram of glycolysis.

**(C-F)** The mRNA expression levels of glycolytic enzymes in BMMNC of AML#1, as determined by RT-qPCR (n = 3 independent experiments).

Data are shown as mean ± SD, by Student's t-test (**P* < 0.05, ***P* < 0.01, ****P* < 0.001).

**Sup Figure 5. 3D bioprinting recapitulates drug tolerance in leukemia cells**

**(A)** Dose-response curves and IC_50_ of 3D-cultured BMMNCs treated with AraC for 24 h (n = 3 technical replicates).

**(B)** Dose-response curves and IC_50_ of 3D-cultured BMMNCs treated with DNR for 24 h (n = 3 technical replicates).
**(C)** BMMNCs cultured in 2D or 3D systems were treated with AraC for 72 h. The percentages of early and late apoptotic cells were determined by flow cytometry (n = 3 technical replicates).
**(D)** BMMNCs cultured in 2D or 3D systems were treated with DNR for 72 h. The percentages of early and late apoptotic cells were determined by flow cytometry (n = 3 technical replicates).
**(E)** BMMNCs cultured in 2D or 3D systems were treated with AraC (AML#2 225 nM, AML#3 140 nM) for 24 h, and cell-cycle profiles were analyzed by flow cytometry (n = 3 technical replicates).
**(F)** BMMNCs cultured in 2D or 3D systems were treated with DNR (AML#2 5 nM, AML#3 6 nM) for 24 h, and cell-cycle profiles were analyzed by flow cytometry (n = 3 technical replicates).

Data are shown as mean ± SD, by unpaired Student's t-test (A-B) or one-way ANOVA (C-F) (**P* < 0.05, ***P* < 0.01, *** *P* < 0.001, *****P* < 0.0001, ns, not significant).

AraC, Cytarabine; DNR, Daunorubicin.

**Supplementary Table**

**Sup Table 1. Sequences of forward and reverse primers used in PCR amplification**

| **Mutation** | **Forward Sequence (5’→ 3’)** | **Reverse Sequence (5’→ 3’)** |
| --- | --- | --- |
| *NRAS* | TGTAAAACGACGGCCAGTCAGAAGTGTGAGGCCGATATT | CAGGAAACAGCTATGACCGATGATCCGACAAGTGAGAGAC |
| *U2AF1* | TGTAAAACGACGGCCAGTGAAAGTCTTATTAAAGCGTGGATGG | CAGGAAACAGCTATGACCGGCACAGGAATACTCACTTCTT |
| *SRSF2* | TGTAAAACGACGGCCAGTTTCGCCTTCGTTCGCTTT | CAGGAAACAGCTATGACCCCGCGGACCTTTGTGAG |
| *DNMT3A* | AGGTGTGTGTTGAGAAGCTGAT | GAGAGCAGGTCATTCAAGTCCT |

**Sup Table 2. Sequences of forward and reverse primers used in RT-qPCR assays.**

| **Gene** | **Forward Sequence (5’→ 3’)** | **Reverse Sequence (5’→ 3’)** |
| --- | --- | --- |
| *ACTIN* | CGAGCGCGGCTACAGCTT | CCTTAATGTCACGCACGATT |
| *TFRC* | ACCATTGTCATATACCCGGTTCA | CAATAGCCCAAGTAGCCAATCAT |
| *ODC1* | TTTACTGCCAAGGACATTCTGG | GGAGAGCTTTTAACCACCTCAG |
| *TXNRD1* | ATATGGCAAGAAGGTGATGGTCC | GGGCTTGTCCTAACAAAGCTG |
| *SQSTM1* | GCACCCCAATGTGATCTGC | CGCTACACAAGTCGTAGTCTGG |
| *NIP7* | ATGCGGCCTTTGACTGAAGAG | TGCAGACGGAAACAGTAGGTG |
| *MRTO4* | TCCAAGCGCGACAAGAAAGT | GTCCACACATTTCCGAAGCTC |
| *TRIB3* | AAGCGGTTGGAGTTGGATGAC | CACGATCTGGAGCAGTAGGTG |
| *SRM* | GTGGTGGCCTATGCCTACTG | CTCCTGGAAGTTCGTGCTCG |
| *NOP16* | GGTTACAGTGTCAACCGAAAGC | GATGTGGGAGCATTCGATCCG |
| *NDUFS1* | TTAGCAAATCACCCATTGGACTG | CCCCTCTAAAAATCGGCTCCTA |
| *NDUFA8* | CCCAACAAGGAGTTTATGCTCT | CACAGTGACGTTTTATCTGCCT |
| *SHDB* | ACAGCTCCCCGTATCAAGAAA | GCATGATCTTCGGAAGGTCAA |
| *UQCR10* | ATCGTGGGCGTCATGTTCTTC | ATGTGGTCGTAGATAGCGTCC |
| *COX5A* | ATCCAGTCAGTTCGCTGCTAT | CCAGGCATCTATATCTGGCTTG |
| *COX7A2* | CTCGGAGGTAGTTCCGGTTC | TCTGCCCAATCTGACGAAGAG |
| *ATP5F1A* | GTATTGCCCGCGTACATGG | AGGACATACCCTTTAAGCCTGA |
| *ATP5PB* | AGGTCCAGGGGTATTGCAG | TCCTCAGGGATCAGTCCATAAC |
| *COQ3* | AAGCTGCGCGTCCCTTAATTT | TCGTCCTGTAGGATTTGAACCAT |
| *PFKP* | GCATGGGTATCTACGTGGGG | CTCTGCGATGTTTGAGCCTC |
| *PKM* | CATGGCTCCTACGGAGAGGT | ACATGGAACGCTTTACCGCAT |
| *HK1* | GCTCTCCGATGAAACTCTCATAG | GGACCTTACGAATGTTGGCAA |
| *PGK1* | TGGACGTTAAAGGGAAGCGG | GCTCATAAGGACTACCGACTTGG |
