# Supplementary material for: A 3D Bioprinted Platform That Maintains the Functional Integrity of Primary AML Cells
Source: Cell Prolif. 2026 Jun 16:e70250. Online ahead of print. doi: 10.1111/cpr.70250 (PMC13325942; doi:10.1111/cpr.70250)
Supplement: Supplementary file 1 — Figure S1: Characterisation of AML cells in 3D culture conditions. (A) Representative fluorescence‐activated cell sorting (FACS) plots showing CD45⁺CD34⁺CD38⁻ LSCs in BMMNCs from the 2D and 3D culture at day 0 and day 6. (B) Representative Sanger sequencing chromatograms showing AML‐associated hotspot mutations (arrowheads) in genomic DNA extracted from BMMNCs of AML#3 after 6 days of 2D or 3D culture. (C) Bright‐field microscopy images of the printed structures. (D) Representative live/dead staining images and assessment of cell viability of BMMNCs‐laden constructs at days 0, 2, 4, 6 and 8 post‐printing (n = 5 technical replicates). Live and dead cells were labelled with Calcein‐AM (green) and PI (red), respectively. Scale bar, 200 μm. Data are shown as mean ± SD, by one‐way ANOVA (D) (**p < 0.01, ns, not significant). LSC, leukaemic stem cell. Figure S2: Heatmaps of MYC target enrichment (A, B) and mTORC1 signalling enrichment (C) based on RNA‐seq data from 3D‐cultured BMMNCs. Figure S3: 3D culture enhances OXPHOS of AML cells. (A) Heatmaps of oxidative phosphorylation enrichment based on RNA‐seq data from 3D‐cultured BMMNCs. (B–G) The mRNA expression levels of respiratory chain complexes I–V and coenzyme Q in BMMNCs of AML#1, as determined by RT‐ qPCR (n = 3 independent experiments). Data are shown as mean ± SD, by Student's t‐test (*p < 0.05, **p < 0.01, ***p < 0.001). Figure S4: 3D culture enhances glycolysis of AML cells. (A) Heatmaps of glycolysis enrichment based on RNA‐seq data from 3D‐cultured BMMNCs. (B) Schematic diagram of glycolysis. (C–F) The mRNA expression levels of glycolytic enzymes in BMMNC of AML#1, as determined by RT‐qPCR (n = 3 independent experiments). Data are shown as mean ± SD, by Student's t‐test (*p < 0.05, **p < 0.01, ***p < 0.001). Figure S5: 3D bioprinting recapitulates drug tolerance in leukaemia cells. (A) Dose–response curves and IC50 of 3D‐cultured BMMNCs treated with AraC for 24 h (n = 3 technical replicates). (B) Dose–response [file CPR-9999-e70250-s001.docx]

**Supplementary files**

Supplementary Figures 1-5

Supplementary Tables 1-2

**Supplementary Figure**

**
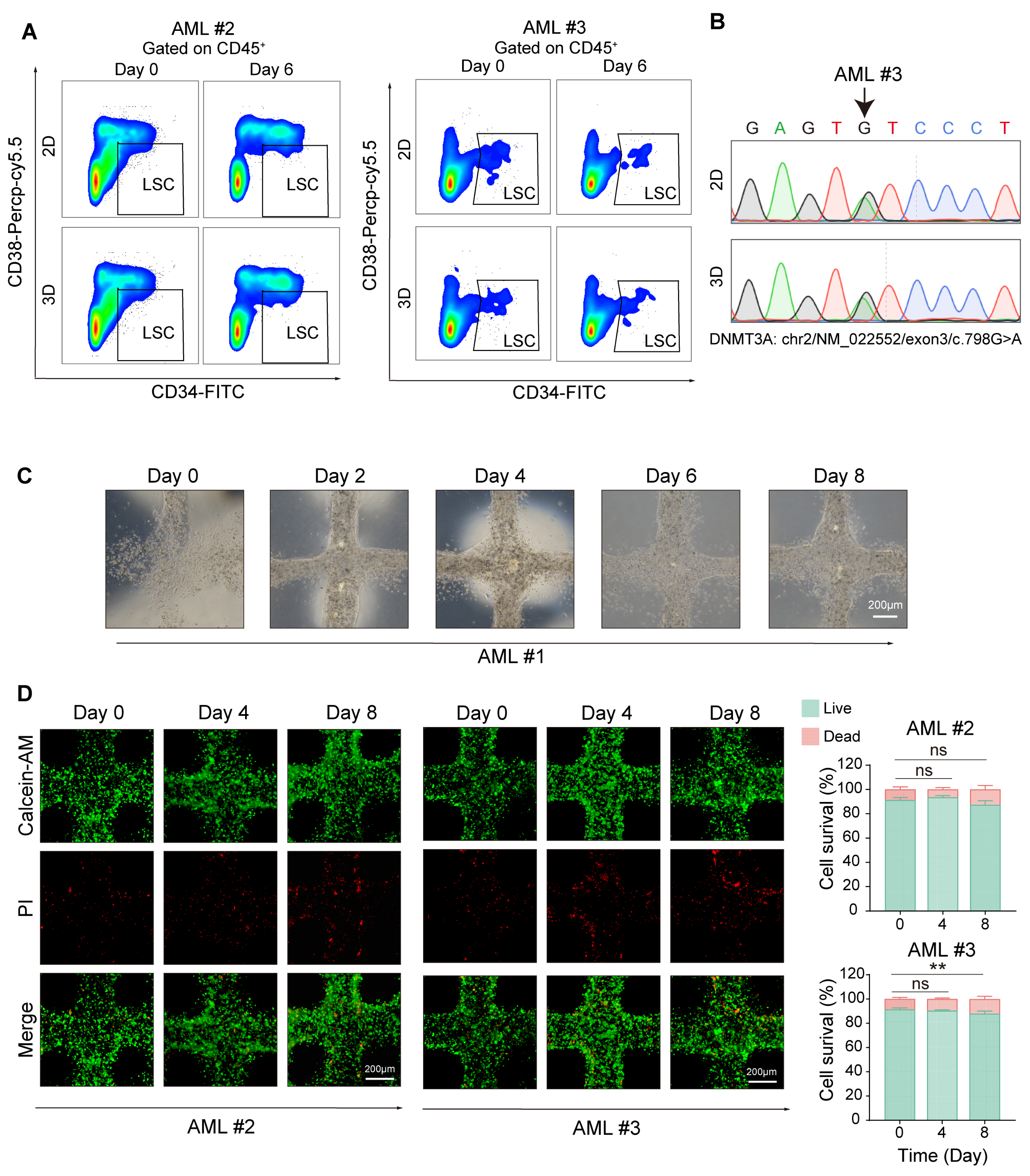
**

**Sup Figure 1.** **Characterization of AML cells in 3D culture conditions**

**(A)** Representative fluorescence-activated cell sorting (FACS) plots showing CD45⁺CD34⁺CD38⁻ LSCs in BMMNCs from the 2D and 3D culture at day 0 and day 6.

**(B)** Representative Sanger sequencing chromatograms showing AML-associated hotspot mutations (arrowheads) in genomic DNA extracted from BMMNCs of AML#3 after 6 days of 2D or 3D culture.

**(C)** Bright-field microscopy images of the printed structures.

**(D)**Representative live/dead staining images and assessment of cell viability of BMMNCs-laden constructs at days 0, 2, 4, 6, and 8 post-printing (n = 5 technical replicates). Live and dead cells were labelled with Calcein-AM (green) and PI (red), respectively. Scale bar, 200 μm.
Data are shown as mean ± SD, by one-way ANOVA (D) (***P* < 0.01, ns, not significant).

LSC, leukemic stem cell.

**
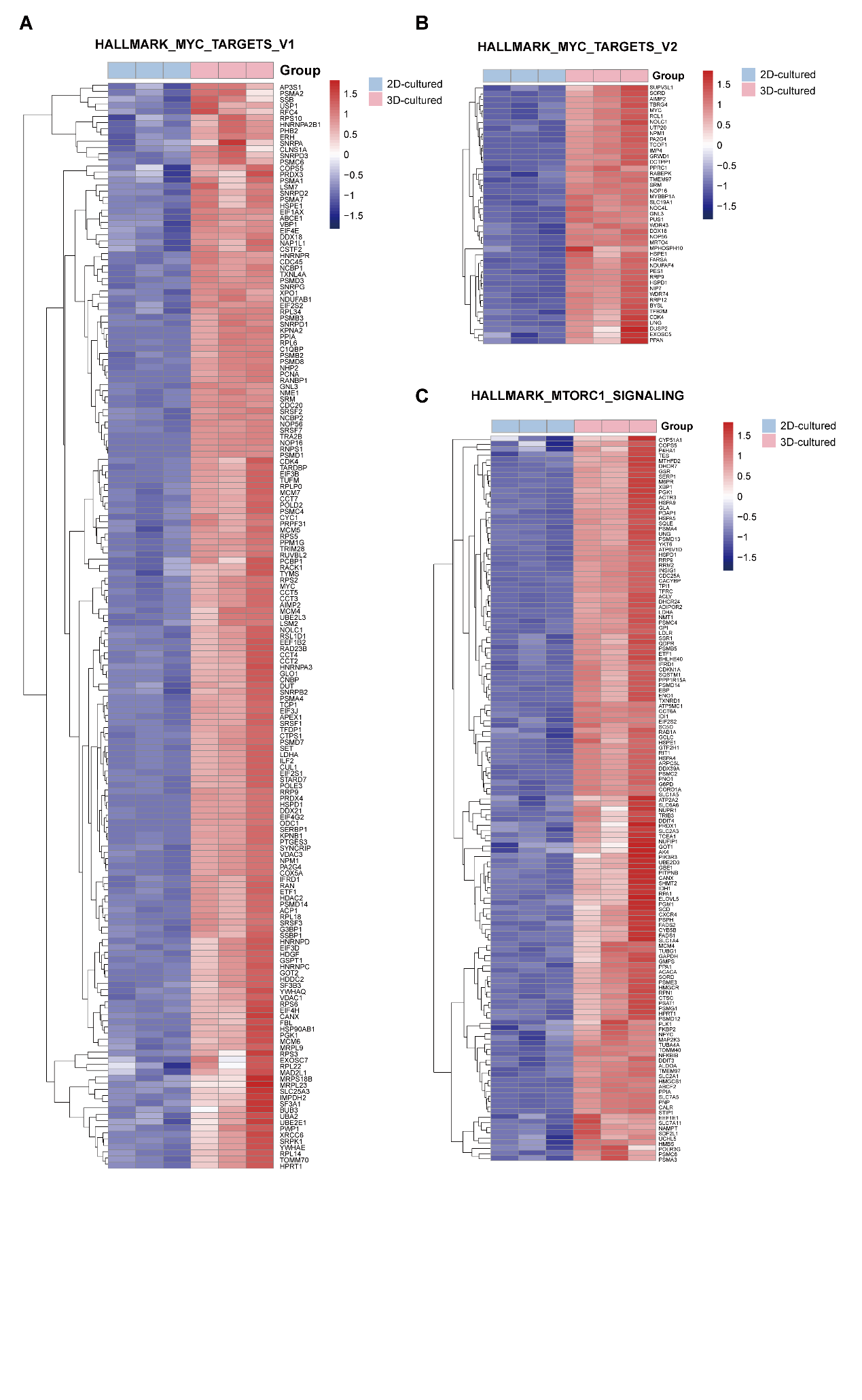
**

**Sup Figure 2.** Heatmaps of MYC target enrichment (A-B) and mTORC1 signaling enrichment (C) based on RNA-seq data from 3D-cultured BMMNCs.

**
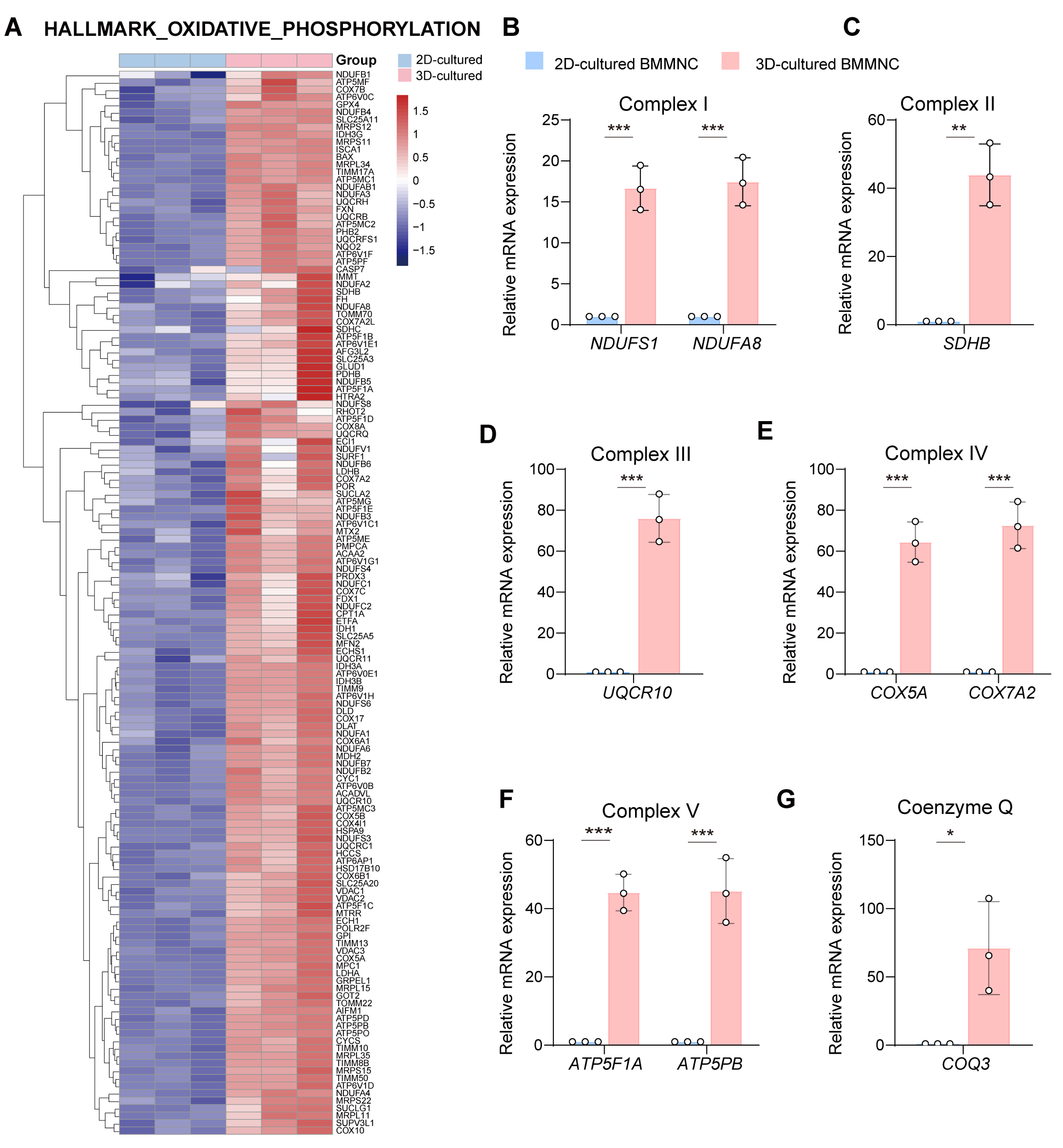
**

**Sup Figure 3. 3D Culture Enhances** **OXPHOS of AML Cells**

**(A)** Heatmaps of oxidative phosphorylation enrichment based on RNA-seq data from 3D-cultured BMMNCs

**(B-G)** The mRNA expression levels of respiratory chain complexes I-V and coenzyme Q in BMMNCs of AML#1, as determined by RT- qPCR (n = 3 independent experiments).

Data are shown as mean ± SD, by Student's t-test (**P* <0.05, ***P* < 0.01, ****P* < 0.001).


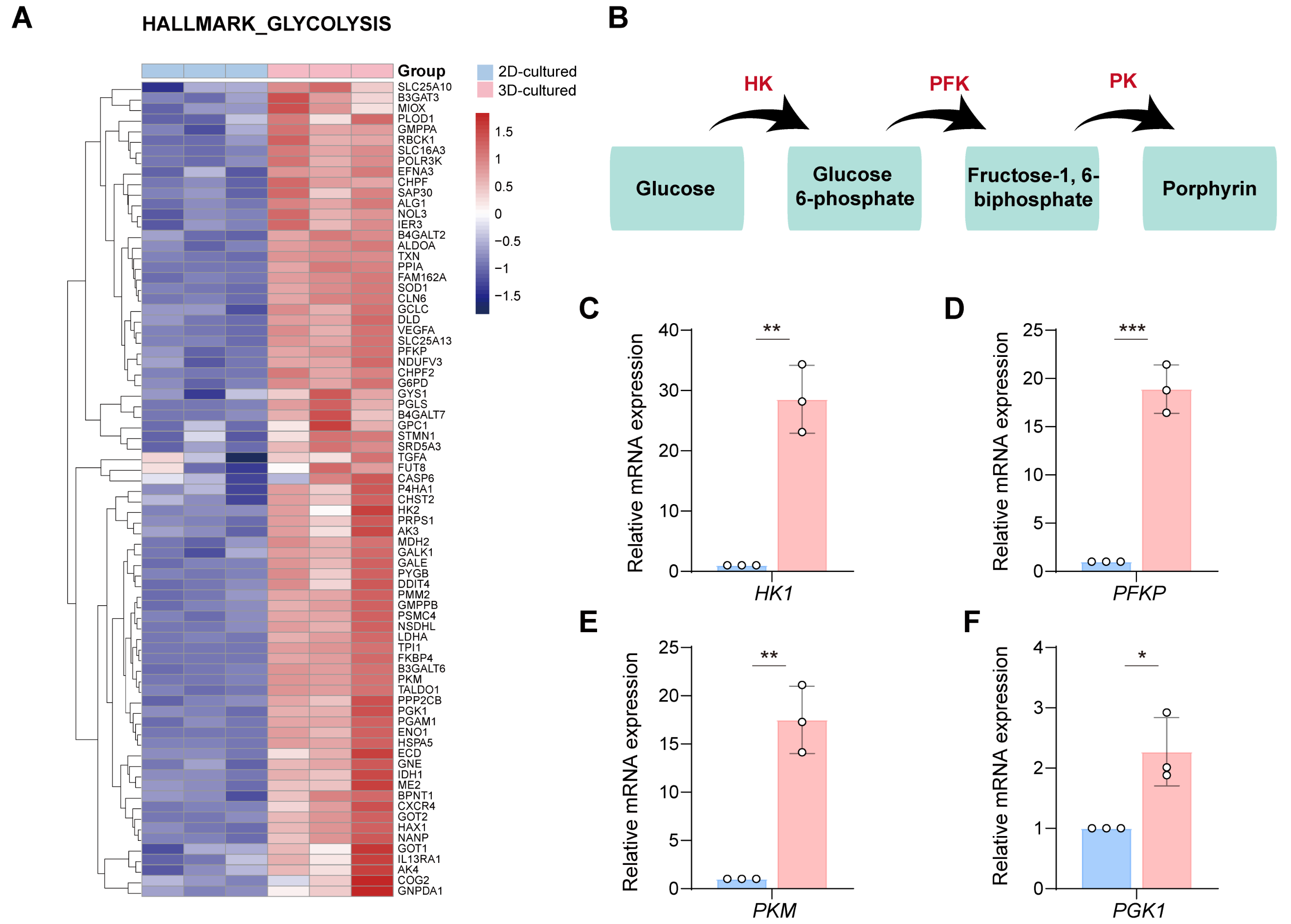


**Sup Figure 4. 3D Culture Enhances glycolysis of AML Cells**

**(A)** Heatmaps of glycolysis enrichment based on RNA-seq data from 3D-cultured BMMNCs(

**(B)** Schematic diagram of glycolysis.

**(C-F)** The mRNA expression levels of glycolytic enzymes in BMMNC of AML#1, as determined by RT-qPCR (n = 3 independent experiments).

Data are shown as mean ± SD, by Student's t-test (**P* < 0.05, ***P* < 0.01, ****P* < 0.001).

**
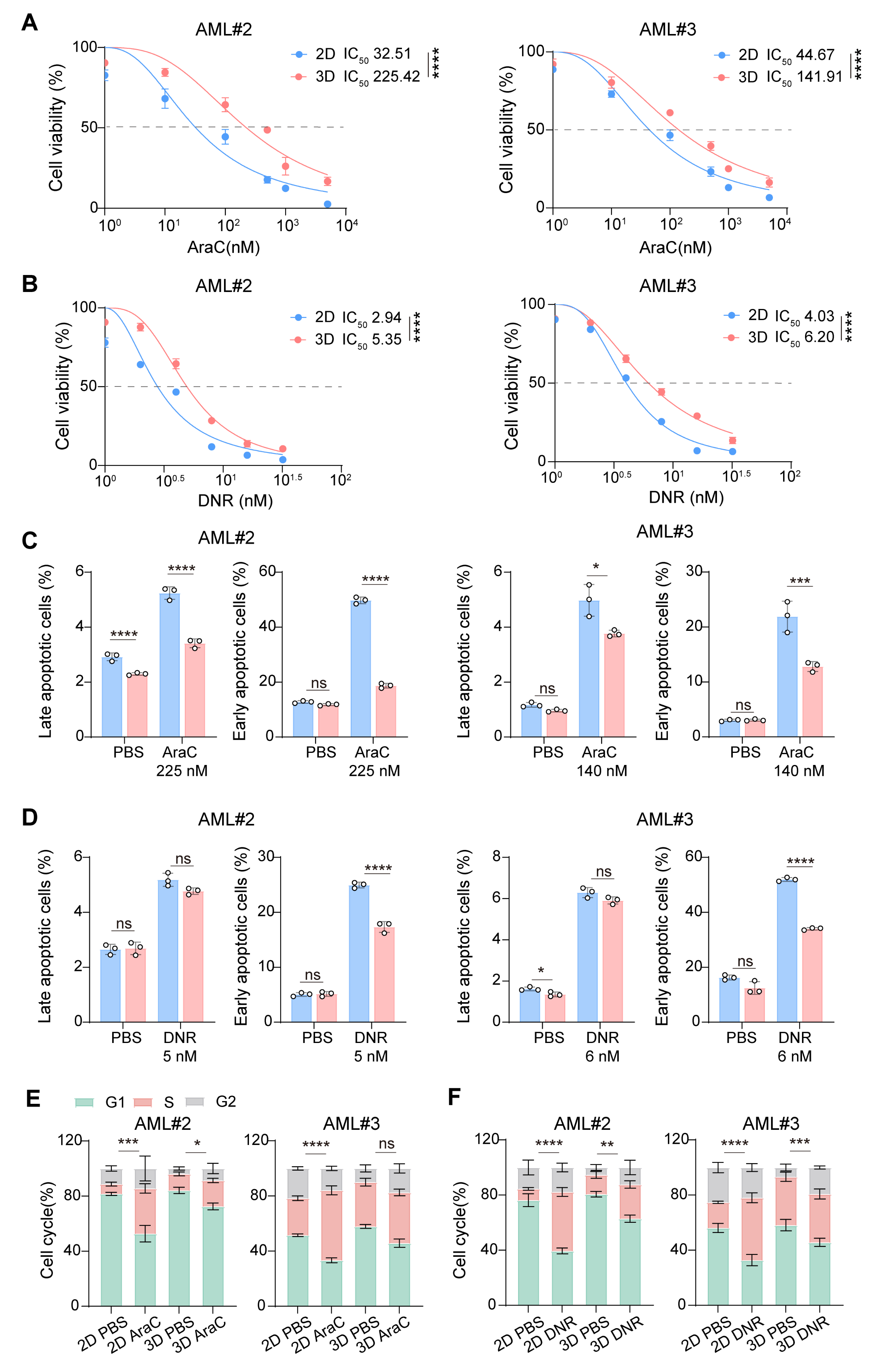
**

**Sup Figure 5. 3D bioprinting recapitulates drug tolerance in leukemia cells**

**(A)** Dose-response curves and IC_50_ of 3D-cultured BMMNCs treated with AraC for 24 h (n = 3 technical replicates).

**(B)** Dose-response curves and IC_50_ of 3D-cultured BMMNCs treated with DNR for 24 h (n = 3 technical replicates).
**(C)** BMMNCs cultured in 2D or 3D systems were treated with AraC for 72 h. The percentages of early and late apoptotic cells were determined by flow cytometry (n = 3 technical replicates).
**(D)** BMMNCs cultured in 2D or 3D systems were treated with DNR for 72 h. The percentages of early and late apoptotic cells were determined by flow cytometry (n = 3 technical replicates).
**(E)** BMMNCs cultured in 2D or 3D systems were treated with AraC (AML#2 225 nM, AML#3 140 nM) for 24 h, and cell-cycle profiles were analyzed by flow cytometry (n = 3 technical replicates).
**(F)** BMMNCs cultured in 2D or 3D systems were treated with DNR (AML#2 5 nM, AML#3 6 nM) for 24 h, and cell-cycle profiles were analyzed by flow cytometry (n = 3 technical replicates).

Data are shown as mean ± SD, by unpaired Student's t-test (A-B) or one-way ANOVA (C-F) (**P* < 0.05, ***P* < 0.01, *** *P* < 0.001, *****P* < 0.0001, ns, not significant).

AraC, Cytarabine; DNR, Daunorubicin.

**Supplementary Table**

**Sup Table 1. Sequences of forward and reverse primers used in PCR amplification**

| **Mutation** | **Forward Sequence (5’→ 3’)** | **Reverse Sequence (5’→ 3’)** |
| --- | --- | --- |
| *NRAS* | TGTAAAACGACGGCCAGTCAGAAGTGTGAGGCCGATATT | CAGGAAACAGCTATGACCGATGATCCGACAAGTGAGAGAC |
| *U2AF1* | TGTAAAACGACGGCCAGTGAAAGTCTTATTAAAGCGTGGATGG | CAGGAAACAGCTATGACCGGCACAGGAATACTCACTTCTT |
| *SRSF2* | TGTAAAACGACGGCCAGTTTCGCCTTCGTTCGCTTT | CAGGAAACAGCTATGACCCCGCGGACCTTTGTGAG |
| *DNMT3A* | AGGTGTGTGTTGAGAAGCTGAT | GAGAGCAGGTCATTCAAGTCCT |

**Sup Table 2. Sequences of forward and reverse primers used in RT-qPCR assays.**

| **Gene** | **Forward Sequence (5’→ 3’)** | **Reverse Sequence (5’→ 3’)** |
| --- | --- | --- |
| *ACTIN* | CGAGCGCGGCTACAGCTT | CCTTAATGTCACGCACGATT |
| *TFRC* | ACCATTGTCATATACCCGGTTCA | CAATAGCCCAAGTAGCCAATCAT |
| *ODC1* | TTTACTGCCAAGGACATTCTGG | GGAGAGCTTTTAACCACCTCAG |
| *TXNRD1* | ATATGGCAAGAAGGTGATGGTCC | GGGCTTGTCCTAACAAAGCTG |
| *SQSTM1* | GCACCCCAATGTGATCTGC | CGCTACACAAGTCGTAGTCTGG |
| *NIP7* | ATGCGGCCTTTGACTGAAGAG | TGCAGACGGAAACAGTAGGTG |
| *MRTO4* | TCCAAGCGCGACAAGAAAGT | GTCCACACATTTCCGAAGCTC |
| *TRIB3* | AAGCGGTTGGAGTTGGATGAC | CACGATCTGGAGCAGTAGGTG |
| *SRM* | GTGGTGGCCTATGCCTACTG | CTCCTGGAAGTTCGTGCTCG |
| *NOP16* | GGTTACAGTGTCAACCGAAAGC | GATGTGGGAGCATTCGATCCG |
| *NDUFS1* | TTAGCAAATCACCCATTGGACTG | CCCCTCTAAAAATCGGCTCCTA |
| *NDUFA8* | CCCAACAAGGAGTTTATGCTCT | CACAGTGACGTTTTATCTGCCT |
| *SHDB* | ACAGCTCCCCGTATCAAGAAA | GCATGATCTTCGGAAGGTCAA |
| *UQCR10* | ATCGTGGGCGTCATGTTCTTC | ATGTGGTCGTAGATAGCGTCC |
| *COX5A* | ATCCAGTCAGTTCGCTGCTAT | CCAGGCATCTATATCTGGCTTG |
| *COX7A2* | CTCGGAGGTAGTTCCGGTTC | TCTGCCCAATCTGACGAAGAG |
| *ATP5F1A* | GTATTGCCCGCGTACATGG | AGGACATACCCTTTAAGCCTGA |
| *ATP5PB* | AGGTCCAGGGGTATTGCAG | TCCTCAGGGATCAGTCCATAAC |
| *COQ3* | AAGCTGCGCGTCCCTTAATTT | TCGTCCTGTAGGATTTGAACCAT |
| *PFKP* | GCATGGGTATCTACGTGGGG | CTCTGCGATGTTTGAGCCTC |
| *PKM* | CATGGCTCCTACGGAGAGGT | ACATGGAACGCTTTACCGCAT |
| *HK1* | GCTCTCCGATGAAACTCTCATAG | GGACCTTACGAATGTTGGCAA |
| *PGK1* | TGGACGTTAAAGGGAAGCGG | GCTCATAAGGACTACCGACTTGG |
